# Orcinol and resorcinol induce local ordering of water molecules near the liquid–vapor interface†

**DOI:** 10.1039/d2ea00015f

**Published:** 2022-08-23

**Authors:** Huanyu Yang, Ivan Gladich, Anthony Boucly, Luca Artiglia, Markus Ammann

**Affiliations:** Laboratory of Environmental Chemistry, Paul Scherrer Institut 5232 Villigen Switzerland markus.ammann@psi.ch; Institute of Atmospheric and Climate Science, ETH Zürich 8092 Zürich Switzerland; Qatar Environment & Energy Research Institute, Hamad Bin Khalifa University P.O. Box 34110 Doha Qatar; Electrochemistry Laboratory, Paul Scherrer Institut 5232 Villigen Switzerland; Laboratory for Catalysis and Sustainable Chemistry, Paul Scherrer Institut 5232 Villigen Switzerland

## Abstract

Resorcinol and orcinol are simple members of the family of phenolic compounds present in particulate matter in the atmosphere; they are amphiphilic in nature and thus surface active in aqueous solution. Here, we used X-ray photoelectron spectroscopy to probe the concentration of resorcinol (benzene-1,3-diol) and orcinol (5-methylbenzene-1,3-diol) at the liquid–vapor interface of aqueous solutions. Qualitatively consistent surface propensity and preferential orientation was obtained by molecular dynamics simulations. Auger electron yield near-edge X-ray absorption fine structure (NEXAFS) spectroscopy was used to probe the hydrogen bonding (HB) structure, indicating that the local structure of water molecules near the surface of the resorcinol and orcinol solutions tends towards a larger fraction of tetrahedrally coordinated molecules than observed at the liquid–vapor interface of pure water. The order parameter obtained from the molecular dynamics simulations confirm these observations. This effect is being discussed in terms of the formation of an ordered structure of these molecules at the surface leading to patterns of hydrated OH groups with distances among them that are relatively close to those in ice. These results suggest that the self-assembly of phenolic species at the aqueous solution–air interface could induce freezing similar to the case of fatty alcohol monolayers and, thus, be of relevance for ice nucleation in the atmosphere. We also attempted at looking at the changes of the O 1b_1_, 3a_2_ and 1b_2_ molecular orbitals of liquid water, which are known to be sensitive to the HB structure as well, in response to the presence of resorcinol and orcinol. However, these changes remained negligible within uncertainty for both experimentally obtained valence spectra and theoretically calculated density of states.

## Abstract 2

Resorcinol and orcinol are surface active in aqueous solution, self-aggregate into islands and induce tetrahedral ordering in nearby water. This may be relevant for ice nucleation promoted by phenolic species in the atmosphere.

## Graphical abstract

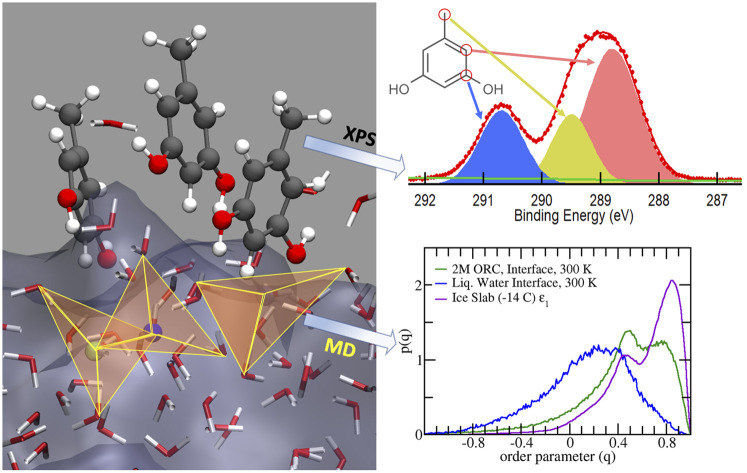

**Environmental significance**

Ice nucleation is one of the key processes in the atmosphere to control cloud occurrence and properties as well as precipitation. Ice nucleation is still poorly understood at the molecular level. Some specific organic compounds are known as particularly good ice nuclei through their ability to order water in their vicinity through patterns of OH groups in a way to facilitate heterogeneous nucleation of ice. Here, we show that orcinol and resorcinol, simple models of a ubiquitously occurring class of organic compounds in the atmosphere, form ordered structures at the liquid–vapor interface of aqueous solutions that induce a higher fraction of tetrahedrally coordinated water molecules in the nearby liquid. This ordering may help to nucleate ice in the atmosphere.

## 1. Introduction

Liquid–vapor interfaces are ubiquitous in the atmosphere, ranging from cloud water droplets, liquid aerosols, to fresh water and oceans. They provide a huge space for phase transfer processes, heterogeneous chemical reactions, and nucleation of solid or other liquid phases. In particular, the nucleation of ice is of paramount importance in atmospheric chemistry and physics.^1^ Freezing of clouds is essential in the formation of precipitation, and ice clouds are covering large parts of the upper troposphere and thus affect the radiative balance of the Earth.^2^ Despite its importance and a large body of literature on experimental and theoretical investigations, the nucleation of ice is still insufficiently understood.

Based on theory and mostly non-linear vibrational spectroscopy experiments, it has been suggested that the local structure of water near ice nucleation (IN) active substrates may be characterized by a comparably large fraction of tetrahedrally coordinated water molecules.^3^ In pure liquid water this would only occur at low temperatures, towards temperatures at which homogeneous nucleation could occur. This property of IN active substrates to order water may be a component involved in heterogeneous nucleation of ice at higher temperature. In the context of organic species in the atmosphere, such ordering effects have been discussed for IN active proteins and organic surfactants,^4,5^ possibly related to a pattern of hydrophilic groups intermitted by hydrophobic moieties in these materials. OH groups are well known to facilitate such ordering effects. For instance, phloroglucinol (benzene-1,3,5-triol), probably one of the most IN active materials overall,^6–8^ in its dihydrate crystal form, exhibits a pattern of hydrophilic OH groups on one of its faces (orthogonal to the stacks of the aromatic rings) that provides close lattice match to ice, while hydrophobic moieties modulate the interaction strength to facilitate water ordering in tetrahedral coordination.^9^ A long record of studies exists on the freezing behavior of long chain fatty alcohols, which is linked to their ability to form ordered 2D structures leading to OH group patterns commensurable with the lattice of ice.^10–19^ The role of such fatty alcohol monolayers as IN active material has also been suggested to be of minor relevance, because the intermolecular forces driving this ordering are relative weak and especially for the complex mixtures of organic material in atmospheric aerosol particles would rarely allow the formation of ordered monolayers or large enough patches.^18^ For fatty alcohols (or related species) the formation of strongly ordered 2D phases is limited to insoluble, long-chain representatives of alcohols. In turn, aromatic compounds are known to undergo π stacking leading to pronounced self-aggregation on surfaces. For instance, such self-aggregation induces structural order among phenol molecules on the liquid water surface and, at the same time, among the hydrated OH groups underneath, which leads to changes in the nearby water structure.^20^ Thus, such surface active aromatic solutes with hygroscopic functional groups such as OH may promote IN, counteracting their effect as solutes that lower the freezing temperature.^21^

Phenolic compounds (aromatic compounds with varying OH substitution) occur as constituents in atmospheric particles in the ng to μg m^−3^ range.^22,23^ Phenolic species and methylated derivatives may be formed from oxidation of aromatic precursors such as benzene,^24^ phenol or cresols.^25^ A large primary source of phenolic species is biomass burning (wildfires or residential fires), in which they constitute a substantial fraction of particulate mass, accompanied by a range of methoxyphenols.^26–28^ The atmospheric lifetime against oxidation is between hours and days,^22^ also allowing long-range transport within the troposphere and into the stratosphere.^29^ Ring retaining oxidation in the gas and aqueous phase leads to further hydroxylation and also to formation of polyphenols, which contribute to mass and light absorption of secondary organic aerosol.^28,30,31^

In this work, we characterize the surface propensity and the orientation of the organic and the hydrogen bonding (HB) structure of water molecules at the liquid–vapor interface in presence of resorcinol (benzene-1,3-diol, RES) and orcinol (5-methylbenzene-1,3-diol, ORC) in water. RES and ORC are simple members of the family of monomeric phenolic compounds. RES has been directly determined, usually along with catechol (benzene-1,2-diol), in biomass burning emissions and in wildfire plumes.^32–35^ ORC and other methyl- and dimethylbenzendiol isomers have been detected in biomass burning emissions as well.^36,37^ When dissolved in aqueous aerosols or cloud droplets, due to their amphiphilic nature with a hydrophobic aromatic ring and hydrophilic OH groups, these molecules exhibit significant propensity for the liquid–vapor interface.^38–40^ RES is substantially less reactive than catechol and therefore better suited for the present study. The distance among the two OH groups is similar to that in the trihydroxybenzene phloroglucinol mentioned above. ORC features an additional methyl group (but identical OH substitution) that leads to lower solubility and higher surface activity. Early studies have addressed the ice nucleation activity of RES^41^ and other related compounds.^42–44^

Liquid jet X-ray photoelectron spectroscopy (XPS) is a powerful surface sensitive technique to probe the liquid–vapor interface within nm depths of the surface.^45–51^ The shallow probe depth, and thus its surface sensitivity, is the result of strong inelastic scattering of photoelectrons. The inelastic mean free path (IMFP), and thus the depth from which they can escape, is on the order of nm and much shorter than the attenuation length of soft X-rays on the order of μm. Because the photoemission intensity is proportional to the sample's atomic density, XPS can provide quantitative elemental ratios and thus gives access to surface composition. Moreover, element oxidation states, chemical bonding and molecular orientation can be assessed.^47,48,51–53^ As the IMFP of photoelectrons depends on their kinetic energy, the tunable photon energy provided by a synchrotron light source offers the possibility of varying the probe depth.^54^ Photoelectron spectroscopy also provides insights on the valence states of aqueous solutions. The valence levels of water are determined by the physical environment (*e.g.*, the hydrogen bonding (HB) structure, or local orientation) and can be used to determine the effect of solute addition on the local HB structure of water.^49^ The difference in the relative photoemission intensity related to the excitation of 1b_1_, 3a_1_ and 1b_2_ molecular orbitals (MO) between liquid water and ice have been (tentatively) associated with changes of the water HB network between the two phases.^55–57^

O K-edge near edge X-ray absorption fine structure (NEXAFS) spectroscopy probes the absorption of electro-magnetic radiation by excitation of core electrons into unoccupied molecular orbitals *via* dipole induced transitions. Similar to the case of valence level photoemission, the O K-edge NEXAFS spectra are sensitive to the local HB network and enable the identification of water that is tetrahedrally coordinated as in ice or in a more disordered configuration as in liquid water.^58–61^ When detected in electron yield mode, NEXAFS provides this configurational information over just a few nanometer of depth beneath the surface, again due to the short IMFP of the electrons detected.

Atomistic simulations based on molecular dynamics simulations can enable the study of physicochemical processes at atomic and molecular resolution, a resolution which is often hardly accessible experimentally. Classical MD relies on a force field, *i.e.*, a set of predefined parameters and functional forms, describing all the inter- and intramolecular interactions. Thanks to that, classical MD can nowadays model systems of thousands of atoms, spanning hundreds of nanometer in extent, and time scales up to microseconds, which makes this type of simulations suitable for studying adsorption and solvation processes.^62,63^ In *first-principle* (FP) MD forces are calculated *on-the-fly* by electronic structure calculations (usually) at density functional theory: FPMDs has propelled and supported studies of chemical reactions and spectroscopy but their computational cost limits the spatial and temporal scales that can be explored.^63–65^

This work combines experimental results with theoretical atomistic simulations to measure and characterize the surface properties of RES and ORC aqueous solutions. Spectroscopic results show the orientation of surfactants and local ordering of water molecules at the liquid–vapor interface. MD simulations and Density of States (DOS) calculations support the interpretation of the experimental findings, quantifying the solvation preference (*i.e.*, bulk *vs.* surface) of ORC and RES and the tetrahedral ordering of interfacial liquid water induced by the two adsorbates.

This work is not directly providing a parameterization to calculate ice nucleation rates in the atmosphere but aims at improving the fundamental understanding of how ice nucleation active materials influence water with which they are in contact.

## 2. Sample and experimental methods

### 2.1 XPS and NEXAFS

RES (benzene-1,3-diol) and ORC (5-methylbenzene-1,3-diol) were purchased from Sigma-Aldrich and VWR-international, respectively. Both were used without further purification. By using Millipore water (conductivity 18.2 MΩ), followed by deaeration with an inert gas (Ar), we prepared 0.01 and 2.0 M solutions of RES, as well as 0.01 M and 0.2 M solution of ORC. The solutions were not adjusted for pH; the p*K*_a_ values of RES and ORC are 9.4 and 9.6, respectively. Thus, both are assumed to remain undissociated. While RES is very soluble (717 g L^−1^ (25 °C)^66,67^), the solubility of ORC is lower (estimated at 16 and 104 g L^−1^ (25 °C)^68^). Therefore, the 0.2 M remains sufficiently below the solubility limit to avoid clogging in the liquid jet nozzle (see below). All solutions contained 0.05 M NaCl to limit the build-up of streaming potentials. To avoid possible photo degradation and oxidation, all sample solutions were prepared within 24 hours before the experiment, avoiding the exposure to light as much as possible.

Reference experiments with ice were performed at the *In Situ* Spectroscopy beamline (X07DB) at SLS using the setup and procedures described by Orlando *et al.*^69^ and as used for the experiments described by Yang *et al.*^70^

The experiments with the liquid RES and ORC solutions were carried out at the Surface/Interface Microscopy (SIM) beamline at the Swiss Light Source (SLS, Paul Scherrer Institut) using the liquid jet XPS endstation, which is coupled to a hemispheric electron analyzer (R4000, ScientaOmicron) with a high pressure pre-lens (HiPP-2, ScientaOmicron).^45^ The solution under investigation was admitted into the experimental chamber by a PEEK capillary, connected to a vertically mounted quartz nozzle with an aperture ranging from 20 to 25 μm, at a flow rate of 0.6 mL min^−1^. The experimental chamber was pumped to a background pressure of around 10^−3^ mbar. After injection, the solution travels with a laminar flow for a few hundred microseconds, corresponding to a few millimeters in length, before it is hit by the photon beam at 90° from the horizontal electron detection axis (see ESI, Fig. S1†). To avoid charging effects upon photoemission, the nozzle assembly is thoroughly grounded. Before entering the chamber, the capillary was surrounded by a cooling jacket to precool the liquid to 277 K to reduce the vapor pressure in the intermediate vicinity of the liquid filament and the load on the pumping system. During the time in vacuum, evaporation of the liquid lowers the temperature of the liquid by a further few K.^71^ Thus the liquid temperature at the measurement position was around 273 K.

The HB exchange dynamics of water molecules is much faster than desorption, and the time for solute diffusion to build up a monolayer on the surface is on the order of μs for the lowest concentration used in this work, 0.01 M.^46,50^ The liquid jet continuously delivers fresh sample so that beam-induced effects are negligible. During the experiment, we made use of linearly polarized light at 0°. Together with the geometric configuration of photon beam, liquid filament and electron detection axis (Fig. S1†), this was considered in the quantitative interpretation of the photoemission peak intensities (ESI, Section 3†), resulting from the excitation of the C 1s and O 1s core levels. For C 1s, we set the photon energy to 448, 560, 660, and 860 eV, while for O 1s, the photon energy was set to 695, 810, 910 and 1100 eV. Thus, the photoelectron kinetic energies, corresponding to the difference between the photon energy and the binding energy of the core level, which are about 290 eV for C 1s and 540 eV for O 1s, were *ca.* 155, 270, 370 and 570 eV, respectively. The kinetic energy of the photoelectron determines the mean escape depth (MED), which is expressed by means of the IMFP (*λ*) of electrons travelling through the liquid. We calculated *λ* by using the SESSA software and its corresponding database.^72^ With our experimental configuration, the cylindrical surface of the liquid jet beam leads to an effective mean escape depth, MED = 2*λ*/π.^50^ Thus, the four kinetic energies correspond to four different MED values of 4.8, 6.8, 8.4, 11.5 Å, respectively. All XPS peaks were fitted by Gaussians after subtraction of a Shirley background. The peak areas were then normalized with respect to the photon flux and the total photoionization cross section (see ESI, Section 3†).

Valence spectra of 2.0 M RES, 0.2 M ORC, liquid water and ice were measured by using 600 eV of photon energy. Such spectra mainly consist of the contributions of the 1b_1_, 3a_1_ and 1b_2_ MOs of water, with their binding energy lower than 20 eV, but also contain contributions by the MOs of RES or ORC. The limitation to the relatively high photon energy (and correspondingly high kinetic energy) was due to the lower photon flux at the ISS beamline used to obtain the spectrum for ice.

The O K-edge NEXAFS spectra of water and aqueous solutions were obtained by integrating the secondary electrons deriving from the oxygen Auger KLL peaks within the kinetic energy range of 412–437 eV at a pass energy of 50 eV, while scanning the excitation photon energy across the O K-edge from 527 to 560 eV. The chosen kinetic energy window remained free of contributions from photoemission peaks within this photon energy range. Acquisition of the electron yield in the kinetic energy range around the Auger peaks themselves would require a complex subtraction of valence level photoemission peaks.

### 2.2 MD simulation

Atomistic simulations based on classical and *first-principle* molecular dynamics (MD) were employed to clarify the solvation preference of ORC and RES, to quantify the tetrahedral ordering of interfacial liquid water molecules induced by the presence of the two adsorbates, and to determine the spectroscopic signature of the investigated solutions. All classical MD simulations were performed using the GROMACS 2018 Molecular Dynamics package.^73^ The CP2K molecular dynamics software^74^ was used to perform the *first-principle* DOS calculations. More details are provided in the ESI (Section 4.3†).

A liquid water slab of ∼1.48 × 1.48 × 7.2 nm^3^ and 216 water molecules with two vapor/liquid water interfaces was equilibrated at 1 bar pressure and 300 K using classical MD. A liquid water slab of similar (or identical) size has been successfully used in the literature to study the interfacial and bulk solvation in slab systems.^75^ Starting from the equilibrated liquid water slab, three different aqueous solutions were prepared. In the first two, 4 ORC (or 4 RES) molecules were placed on each of the two liquid water interfaces, for a total of 8 ORC (or 8 RES) in the system and a 2 M concentration solution. In the third configuration (0.2 M), one ORC was placed on one interface. Note that upon equilibration, due to partitioning of ORC or RES to the interfaces, the local concentrations in the bulk were reduced. In contrast, in the experiments, the bulk concentration of the equilibrated system remained 2.0 M and 0.2 M for RES and ORC, respectively. Due to the lower solubility of ORC the 2.0 M ORC case could not be studied experimentally. Nevertheless, these three model systems reasonably reflect the experimental conditions and, at the same time, offer a molecular picture of the dynamics of the two compounds in a similar concentration range, which appear beneficial to highlight physicochemical details of the adsorption and interfacial water ordering for the aqueous solutions of interest here.

As described in more detail in the ESI (Section 4.3†), force field parameters for ORC and RES were created according to GAFF2 practice.^76^ Water molecules were described using two different water models: TIP3P,^77^ which is the reference model for the GAFF2 force field, and TIP4P/2005,^78^ which is one of the most commonly used water models for ice and supercooled liquid water.^79^ Since TIP3P is the reference model for GAFF2, simulations employing the GAFF2/TIP3P combination were adopted to describe the surface *vs.* bulk preference of RES and ORC. To the best of our knowledge, there are no force fields for small organics available in the literature to work with TIP4P/2005. However, TIP4P/2005 is superior in describing the water phase diagram and the HB network in the ice phase and in the supercooled region.^79^ A detailed analysis of TIP4P/2005 liquid water and ice structures is available in previous work.^80^ Therefore, simulations with TIP4P/2005 were only used to quantify the tetrahedral ordering of interfacial liquid water molecules and the spectroscopic signature of the system in the presence of the two adsorbates. We run a 400 ns constant volume and temperature (NVT) classical MD at 300 K for each of the three solutions (*i.e.*, 0.2 M ORC, 2 M ORC, and 2 M RES) and for each of the two water models, for a total of six independent MD runs.

We performed additional 400 ns MD simulations of a hexagonal ice (*i.e.*, Ih ice) slab. Ice Ih is the most stable crystalline form of ice in the atmosphere. The ice slab comprised of 320 TIP4P/2005 water molecules with two equilibrated basal ice interfaces exposed to the vapor phase (see ESI Fig. S5†). The *I*_h_ ice slab was equilibrated at *T* = 237 K, which corresponds to 14 K below the melting temperature (*T*_m_ = 251 K) of TIP4P/2005.^81^ An order parameter, *q*_*i*_, was adopted to quantify the tetrahedral arrangement of each water molecule with respect to its (four nearest) neighbors.^80,82,83^1
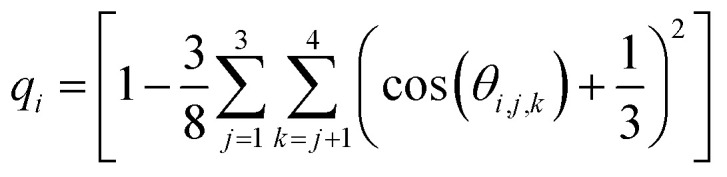


In eqn (1), the sums run over the four nearest oxygen atoms of the oxygen belonging to the water molecule *i*. The angle *θ* is the angle between oxygens *i*, *j*, and *k*, with the oxygen *i* as the angle vertex. In the interior of a perfect ice crystal, *q*_*i*_ = 1 since the four nearest neighbors are tetrahedrally arranged around the *i*th oxygen. In real ice, deviations from the perfect tetrahedral order occur due to thermal motion and defects in crystal structure, giving rise to a relatively narrow distribution of values with a maximum close to *q*_*i*_ = 1. In the liquid water phase, *q*_*i*_ values significantly smaller than 1 are expected since the tetrahedral arrangement of water molecules is distorted in the liquid phase. Following the strategy of Conde *et al.*,^84^ Gladich and Roeselova^80^ identified a threshold value of *q*_t_ = 0.9054 for TIP4P/2004 which discriminates between “ice-like” or “liquid-like” water molecules: for *q*_*i*_ > *q*_t_ the *i*-water molecule belongs to the ice phase, while for *q*_*i*_ < *q*_t_ it belongs to the liquid. Probability distributions for *q* were calculated for all the simulation systems described above, and also for one extra simulation box at ∼0.2 M ORC (*i.e.*, 5400 TIP4P water and 24 ORC) to support our results with a larger simulation box case.

The electronic density of states (DOS) for the different aqueous solutions and ice systems were computed at *first-principles* level employing calculations with SCAN^85^ density functional theory (DFT). The TZV2P basis set with a cutoff of 600 Ry was employed for the valence electrons, while core electrons were modeled using pseudopotentials optimized for SCAN. SCAN is a recently introduced DFT method, designed especially for the electronic structure of water and dynamic simulations.^85^ DOSs were averaged over 160 snapshots extracted every 2 ns from the last part of the classical MD trajectories adopting the TIP4P/2005 water model. A Gaussian smearing of 0.5 eV was used to smooth the DOS. The averaged DOS was then compared with the experimental X-ray photoemission spectra of the valence levels: a similar strategy has already been successfully adopted in the literature for the comparison of the computational DOS with XPS data from liquid water or soft matter systems.^86–90^ DOSs for bulk ice (*i.e.*, with no interfaces) and for an ice slab with two vapor/ice interfaces (Fig. S6†) were calculated at the same temperature of the ice slab MD (*i.e.*, *T* = 237 K).

Further details about the preparation and equilibration of the liquid water and ice slab, the force field strategy, and other computational settings are reported in the ESI.†

## 3. Results and discussion

### 3.1 Surface orientation and surface propensity of RES and ORC in aqueous solutions

**Fig. 1:**
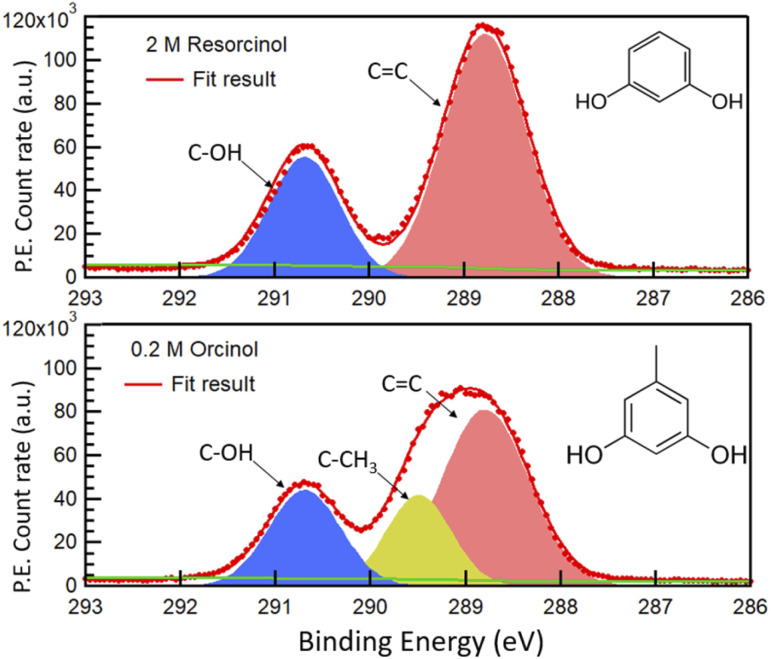
C 1s photoemission count rate of 2.0 M RES (top panel) and 0.2 M ORC (bottom panel) solution, probed with an excitation photon energy of 448 eV. The resulting photoelectrons have a kinetic energy of *ca.* 155 eV. The green line represents the Shirley background. Hydroxy group carbon, aromatic carbon and aliphatic carbon (only for orcinol) contributions are represented by blue, red and yellow shaded Gaussian peaks, respectively.

Fig. 1 shows the photoemission signal of C 1s of 2.0 M RES and 0.2 M ORC solutions, probed with an excitation photon energy of 448 eV, which corresponds to a photoelectron kinetic energy of 155 eV, thus the most surface sensitive mode. The spectra were fit with contributions related to hydroxy group (alcoholic) carbon (solid blue, 290.7 eV), aromatic carbon (solid red, 288.5 eV) and aliphatic carbon (solid yellow, 289.2 eV), all with equal full width at half maximum (FWHM). The O 1s spectra of these two samples at the same MED are reported in Fig. S2.† Apparently, the 0.2 M ORC solution leads to similar C 1s intensity as the 2.0 M RES solution, in spite of the lower bulk concentration (the *y*-axis scales in Fig. 1 are the same). Therefore, these spectra directly show that ORC is substantially more surface active than RES. The surface coverages will be quantified further below.

**Fig. 2:**
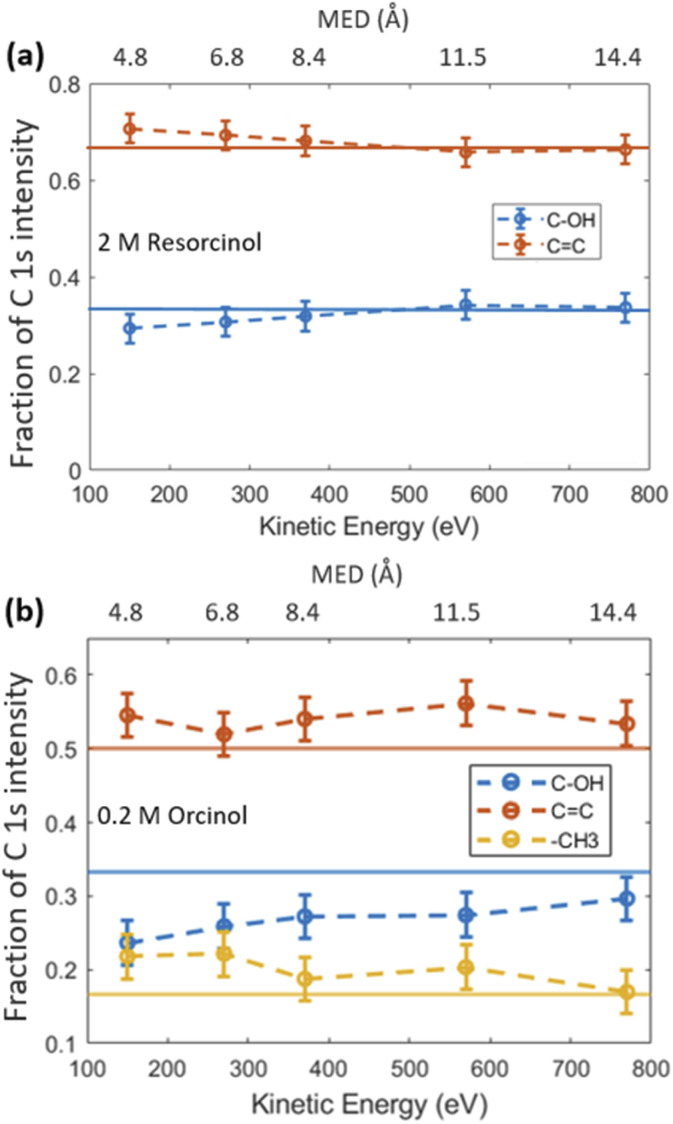
(a) The fractional contribution of aromatic carbon (–C=C, red) and carboxylic carbon (=C–OH, blue) components to the C 1s photoemission intensity in 2 M RES solution. The blue and red thin lines indicate the theoretical ratio of the carboxylic carbon (33.3%) and aromatic carbon (66.7%), respectively. (b) Fractional contributions of carbons for the 0.2 M ORC case, including the aliphatic carbon contribution (–CH_3_, yellow).

The fractional contributions of each C 1s component, derived from the spectra at different kinetic energies, are shown in Fig. 2. Each kinetic energy corresponds to a specific MED, and the photoemission intensity represents the integral of exponentially decreasing contributions from atoms with depth, but with 80% of the signal originating from the topmost layer with thickness equal to the MED. Thus, for the most surface sensitive measurement with MED of 4.8 Å, Fig. 2 gives information about the integrated contributions of specific carbon atoms from the top down to 4.8 Å (80% of the signal). Correspondingly, for larger MED, the signals represent the integrated contributions from the top down to, *e.g.*, 14.4 Å (most bulk sensitive measurement). In the case of 2.0 M RES (Fig. 2a), at low kinetic energy, the contribution of the C–OH carbons to the total C 1s intensity is lower than expected based on the molecular structure (1/3), while the C=C contribution is higher than expected (2/3). At higher kinetic energy, the contributions match the expected values. This implies a preferred orientation of resorcinol molecules residing at the surface: the aromatic rings have an upward orientation with respect to the solution–air interface (see schematic illustration in ESI Section 3, Fig. S3†), due to the preferential hydration of the OH groups and the hydrophobic interaction of the remainder of the molecule. The slightly enhanced photoemission signal intensity contribution comes from the fact that photoelectrons from the C=C carbons, which are located on the vacuum side of the interface, are less attenuated than those from the C–OH carbons. The C 1s spectrum of the ORC solution contains three components (Fig. 1b): C=C, C–OH and –CH_3_, with carbon number ratio 3 : 2 : 1, respectively. We observe an even stronger effect of surface orientation than in the case of RES, with quite significantly opposing behavior of –CH_3_ and C–OH carbons as a function of kinetic energy, consistent with the OH groups pointing towards the solution-side, and the methyl group towards the vacuum-side of the interface. The reason for the apparently less pronounced impact of orientation on the C 1s component fractions for RES than for ORC is that the bulk concentration of RES was much higher than that of ORC: thus, averaging over an already small probe volume was sufficient for the signal to be dominated by the bulk phase molecules.

**Fig. 3:**
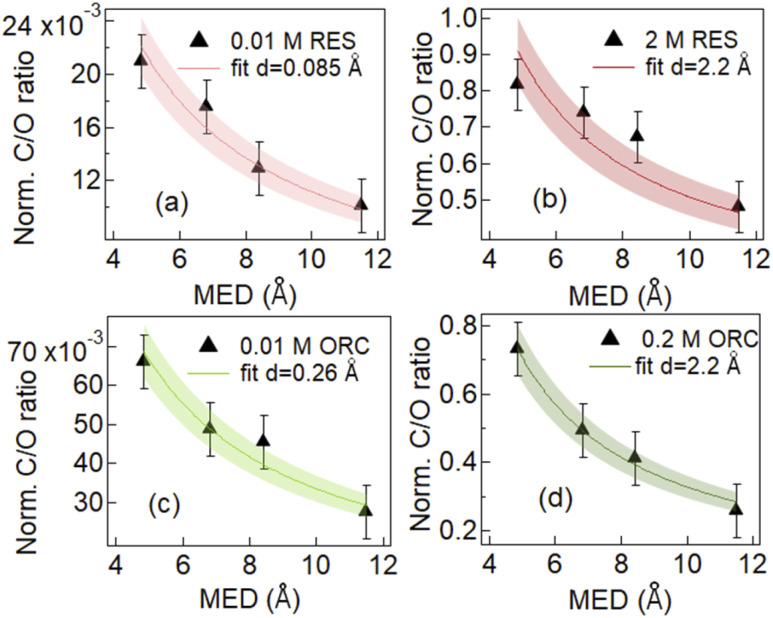
Evolution of normalized C/O photoemission intensity ratio as a function of mean escape depth (MED) for aqueous solutions of 0.01 M RES (a), 2 M RES (b), 0.01 M ORC (c) and 0.2 M ORC (d). Red and Green lines represent the fitting result for resorcinol and orcinol, with one standard deviation shown by the shaded area. The effective thickness *d*, which was the only fitting variable, obtained from the fit is attached in the image legend.

In the following we investigated the surface excess and the effective thickness of the layer established by the RES and ORC molecules on the surface. We first determined the ratio of the C 1s and O 1s signal intensities, depicted in normalized form in Fig. 3. If an element is homogeneously distributed with depth, such as that of oxygen of liquid water, the signal intensity increases linearly with the MED (after normalization to take into account the cross section and photon flux). If an element just exists at the surface, the normalized signal intensity remains constant with increasing MED. Therefore, the ratio of the latter to the former shows a decreasing profile with increasing MED.^91^ Hence, the shapes of these ratios depicted in Fig. 3 indicate the surface activity of RES and ORC. We again note the cumulative character of the XPS signal, with the exponentially decaying contributions from atoms with depth, with the integration starting at the top of the surface for each kinetic energy. In addition, contributions of the solutes in the bulk contribute to the total C 1s intensity as well. We therefore used an attenuation model explained in the ESI and Fig. S3† for a quantitative analysis of these ratios. Briefly, we replace the complex arrangement of carbon atoms of the RES and ORC molecules on the surface by an equivalent thin and homogeneous layer of liquid RES and ORC, respectively, on top of the water. This enables a simple attenuation model to be applied. The density of the water is assumed to feature a rectangular profile below the organic layer. The detailed description of the model and derivation of equations is provided in the ESI (Section 3†).

Fig. 3 shows the results of fits to the measured intensity ratios normalized by photon flux and total differential ionization cross section (abbreviated as normalized C/O ratio), to determine the equivalent thickness of the organic layer (*d*) for 0.01 M and 2.0 M RES and 0.01 M and 0.2 M ORC solutions (see ESI, Section 3†). The results confirm that the investigated solutes clearly exhibit surface propensity. Traditionally, this can be expressed by their surface excess. The surface excess, *Γ*, can be estimated from the product of the surface layer thickness (*d*) and the molecule number density within this layer (*n*_s_: in molecules per cm^3^, reported in Table S1†):

2 *Γ* = *d* × *n*_s_

The geometric dimension of an aromatic ring is about 2.4 Å. In case of 0.01 M RES and ORC 0.01 M solutions, the obtained surface layer thickness, *d*, is 0.085 Å and 0.26 Å respectively. This implies that the surface concentrations were much below a monolayer coverage. Our analysis does not consider whether they randomly float on the surface or aggregate into islands. The effective thickness only reflects the amount of carbon thought to be evenly distributed over the surface and that leads to the average attenuation of the signals observed by XPS. The *Γ* values derived for the dilute solutions are 5.8 × 10^12^ (±8.7 × 10^11^) molecules per cm^2^ and 1.6 × 10^13^ (±2.4 × 10^12^) molecules per cm^2^ for 0.01 M RES and 0.01 M ORC, respectively. As the concentration of the solutions increases to 2.0 M and 0.2 M for RES and ORC, respectively, the surface layer thickness *d* increases to 2.2 Å, which matches almost perfectly the geometric dimension of these organic molecules, suggesting the accumulation of one monolayer at the surface. The corresponding *Γ* values are 1.5 × 10^14^ (±2.3 × 10^13^) molecules per cm^2^ and 1.4 × 10^14^ (±2.1 × 10^13^) molecules per cm^2^ respectively. We note that the model assumes that the RES and ORC concentrations remain constant in the bulk liquid, as accumulation on the surface does not lead to significant depletion in the near surface bulk due to fast enough diffusion from the reservoir of the deeper lying layers. The reported surface excess values are similar to those for phenol and cresol.^92^

**Fig. 4:**
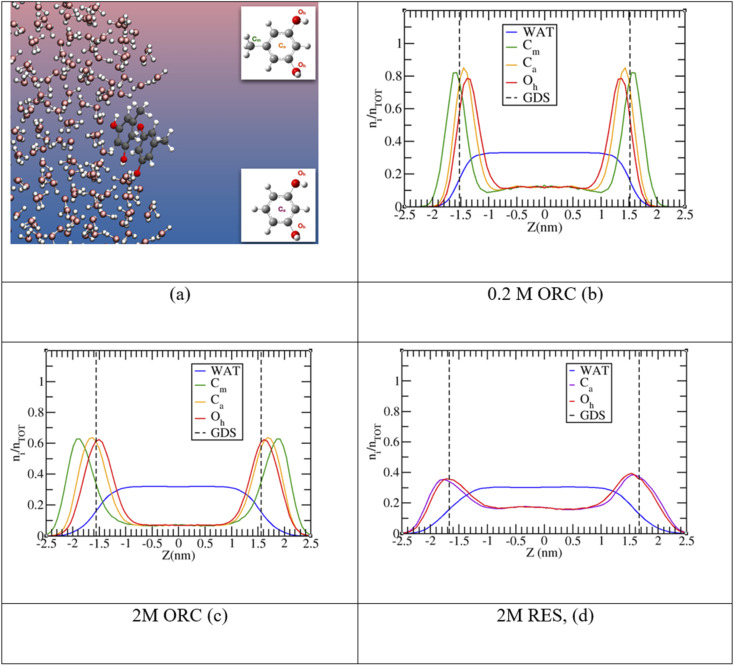
Panel (a): snapshot from a 2 M ORC molecular dynamics trajectory. In the insets resorcinol and orcinol molecules with atom nomenclature: C_m_ (green) is the carbon of the methyl group, C_a_ (orange and violet for ORC and RES, respectively) the center of the aromatic rings, and O_h_ (red) the oxygen of the hydroxyl groups. Panels (b), (c) and (d) show the probability distribution profile, *n*_i_/*n*_TOT_, normalized to unity as a function of the *z*-coordinate perpendicular to the vapor/liquid water interface, obtained collecting the *z*-position of each species over the MD trajectory in TIP3P water. Vertical dashed lines report the Gibbs Dividing Surface (GDS) for the aqueous slab.

The experimental picture for the surface orientation and bulk *vs.* surface solvation preference can be rationalized by looking at the density profile of ORC and RES from the classical MD simulations in Fig. 4. In addition, Fig. S4 in the ESI† shows a snapshot from the 2 M ORC MD simulation and Fig. S5† the solvation preference of ORC and RES in both water models. As mentioned in the methods section, the solvation preference of ORC and RES is better described by the simulations in TIP3P water. Nevertheless, in Table S2 and Fig. S5† we compared the predictions for the solvation preference of ORC and RES using TIP3P and TIP4P/2005: simulations with TIP4P/2005 reported a more marked hydrophobicity for ORC and RES than simulations with TIP3P. While quantitative differences are present, the conclusions drawn about the surface and bulk propensity of ORC and RES are identical between the two water models, showing ORC more surface enhanced than RES.

In agreement with the experimental picture, the computed density profile for ORC (orange) and RES (violet) states that the two molecules have different bulk and surface propensity: in Fig. 4d the RES profile samples more the bulk region than 0.2 M or 2.0 M ORC in Fig. 4b and c, respectively. From the density profiles in Fig. 4, it is possible to calculate the solute surface excess at the Gibbs Dividing Surface, *Γ*, and the bulk concentrations *n*_b_,^93–95^ as detailed in the ESI.† As reported in Table S3,† the calculated *Γ* is 1.2, 1.4 and 0.8 ×10^14^ molecules per cm^2^ for 0.2 M ORC, 2 M ORC, and 2 M RES aqueous solution, respectively. *n*_b_ is 1.8, 1.2 and 2.8 × 10^20^ molecules per cm^3^ for 0.2 M ORC, 2 M ORC, and 2 M RES aqueous solution, respectively. As mentioned in the methods section, equilibration leads to depletion of the bulk density, in contrast to the experimental case, where the bulk concentration remains essentially unaffected. Thus, the values for *Γ* from the simulation should not be quantitatively compared to those from XPS, even though they match to within 30%.

**Fig. 5:**
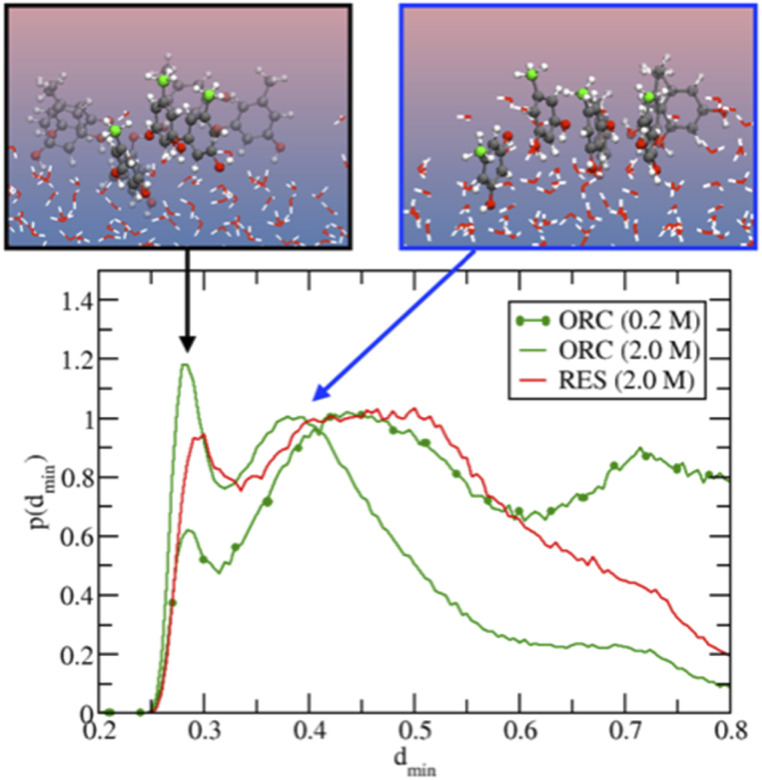
Probability distribution for the minimal intermolecular distances among the hydroxyl oxygens of RES (or ORC) in solution. In the insets, two representative snapshots corresponding to the peaks of the 2 M ORC solution. The carbon atoms of the methyl groups are highlighted in green. For visualization purposes, the distributions were normalized to have the intensity of the peak at ∼0.4 nm equal to unity. The 0.2 M ORC (green and dotted) curve was calculated over a 400 ns MD using a larger water box (5400 TIP4P/2005 water and 24 ORC).

MD simulations also show (Fig. 4) that RES is more present in the bulk of the solution than ORC, even though it still displays a marked hydrophobic character. The difference between the surface propensity of ORC and RES can be rationalized by the presence of the methyl group in ORC, which further pushes the molecule toward the vapor side of the interface: a similar behavior has been observed also for methylamine at the liquid water surface, with the methyl group sticking out from the interface.^96^ The hydrophobicity of the aromatic groups forces ORC and RES to be preferentially solvated at the interface, while the hydroxyl –OH groups are pointing toward the condensed water phase, in agreement with the conclusions from the experiments (Fig. 2). Fig. 4 shows the density profiles for the hydroxyl oxygens (in red) closer to the water interface than the aromatic ring, while the methyl group ones are oriented toward the vapor phase. Similar features have been reported by MD for the phenol (2.5 M) aqueous solution density profile.^20^ Finally, it is interesting to note the formation of π-stacking among the aromatic rings, as shown in a representative snapshot in Fig. 5: similar behavior with π- or slightly parallel displaced stacking has been reported in combined experimental and simulation works in the literature for benzene, naphthalene and methylnaphthalene on the surface of ice and liquid water.^20,97–99^ As mentioned above, the self-aggregation of RES or ORC into islands *via* such π-stacking cannot be assessed by the XPS signals.

Fig. 5 provides further details on the ORC and RES interfacial self-stacking, reporting the probability distribution for the minimal intermolecular distances among the hydroxyl oxygens of RES (or ORC). Fig. 5 shows a bimodal distribution with two peaks, one at ∼0.3 nm and a second one at ∼0.4–0.5 nm. These two peaks are associated to π- or slightly parallel displaced stacking, as shown in representative snapshots for the distribution peaks reported in the insets of Fig. 5. Interestingly, by increasing the solution concentration, the peaks become sharper and the 0.3 nm one also more intense, as shown by comparing the 0.2 M and 2.0 M ORC curves. Note that the distance between the OH groups on the basal plane of ice is also ∼0.3 nm, which is also similar to the hydroxyl group distance in the phloroglucinol dihydrate crystal (0.34 nm),^100^ one of the most IN active material.^9^ Moreover, at the same 2 M concentration, the peaks for the RES solution are much broader than those of ORC (red and green straight lines, respectively): RES samples more the subsurface and bulk region of the solution than ORC, avoiding the interfacial self-stacking. Finally, it is worth mentioning that our classical MD reports a preference for slightly parallel displaced stacking rather than perfect π-stacking of the benzene rings: the difficulty to perfectly model π–π interactions among aromatic rings is well-known in classical MD simulation based on point charges.^99,101^

### 3.2 Hydrogen bonding structure from O K-edge NEXAFS

**Fig. 6:**
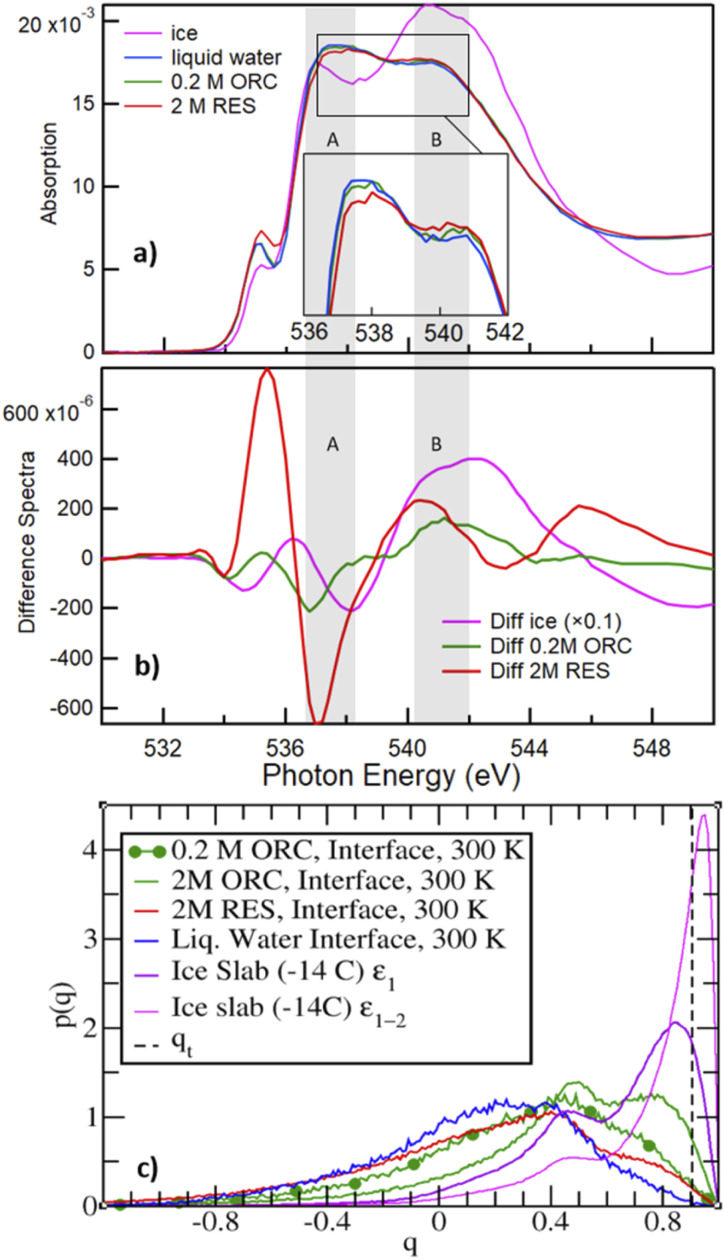
Panel (a) shows the O K-edge NEXAFS of liquid water (blue), ice (magenta), RES 2 M (red), and ORC 0.2 M (green) solution. The inset shows the spectra for water, ORC and RES at an expanded *y*-scale for comparison. Panel (b) shows the difference spectra, obtained by subtracting the liquid water spectrum from the spectrum of ice (black), RES 2 M (red), and ORC 0.2 M (green). The grey shaded regions A and B denote absorption due to weak asymmetric and symmetric tetrahedral HB configurations, respectively. Panel (c) probability density distribution of the tetrahedral order parameter *q* (eqn (1)), for different aqueous solution from MD. *q*_t_ = 0.9054 (vertical dashed line) discriminates between “ice-like” (*q* > *q*_t_) and “liquid-like” water molecules (*q* < *q*_t_), see ref. 51. The interfacial region for the liquid water slab at 300 K was defined in the density profile of Fig. S5.† The *ε*_1_ or *ε*_1_ + *ε*_2_ (*i.e.*, *ε*_1–2_) bilayers defined the interfacial ice region (Fig. S5† and ref. 74). The 0.2 M ORC (green and dotted) curve was calculated over 400 ns MD using a larger water box (∼5400 TIP4P/2005 water and 24 ORC).

Fig. 6a shows the O K-edge NEXAFS spectra of liquid water (blue) and ice (black), compared with those of 2.0 M RES (red) and 0.2 M ORC (green). We distinguish two main absorption regions, marked by the shaded areas A and B: in liquid water, the strong intramolecular covalent O–H bonds in asymmetric weak HB configuration prevail, leading to an enhanced absorption at 537 eV (shaded region A). In contrast, in ice, the predominant strong and highly symmetric tetrahedral HB configuration leads to a prominent absorption at around 541–542 eV (shaded region B), whereas the absorption in region A is quenched due to the shortage of asymmetric HB. The difference spectrum between ice and water NEXAFS shows a negative value in region A and positive in region B (Fig. 6b, pink), consistent with the formation of a more ordered HB structure. The absorption feature at 535 eV can be assigned to free OH groups, which is more prevalent in aqueous solutions (especially also at the solution–vapor interface) than in ice.^102,103^ The contrasting HB configuration embodied by NEXAFS spectra of liquid water and ice serves as a reference to qualitatively interpret the local HB structure of water on the surface of 2.0 M RES (red, Fig. 6a) and 0.2 M ORC (green, Fig. 6a) solutions. Compared to liquid water, the O K-edge NEXAFS spectra indicate a trend towards lower intensity in region A and higher intensity in region B (Fig. 6a) for both RES and ORC solutions. We note that the mean escape depth of the electron yield NEXAFS spectra is not as well defined as with XPS. The kinetic energy window used for the NEXAFS spectra of 412–437 eV is quite far away from the primary Auger peaks, so that electrons contributing to the electron yield may have undergone several scattering events and thus originate from deeper in the bulk than the MED corresponding to this kinetic energy. This leads to a lowering of the influence of the surface region to the NEXAFS spectra. We may also assess the contribution from oxygen atoms of the OH groups of ORC and RES. For the higher coverages obtained from the XPS data, the number density of these oxygen atoms is about one order of magnitude smaller than that of oxygen atoms within the first bilayer of water molecules. Taking the actual probe depth, the contribution of the OH groups themselves to the NEXAFS spectrum and specifically to the ratio in regions A and B is likely negligible. In summary, the observed trends suggest that HB of water molecules near the surface of these organic solutions is slightly more ordered than that in liquid water. We note that in general, solutes in the bulk aqueous phase tend to increase the presence of weak HB configurations due to the effect of hydration, *e.g.*, for salt solutions.^104^ Thus, for that effect we would rather expect an increase in region (A).

The molecular picture for the local arrangement of interfacial water molecules in contact with ORC and RES can be obtained from the classical MD simulations using the tetrahedral order parameter defined in eqn (1). Fig. 6c reports the order parameter probability distributions for interfacial water molecules, *p*(*q*), where the interfacial region is defined for *z* larger (*i.e.*, toward the gas phase) than the Gibbs Dividing Surface. Following Gladich *et al.*,^105^ the interfacial region of the ice slab was determined by the interfacial *ε*_1_ or *ε*_1_ + *ε*_2_ bilayers, outlined in Fig. S6.† As already noted in the literature,^105,106^ at the vapor/pure liquid water interface the tetrahedral arrangement of water molecules is disrupt. This feature is also apparent from Fig. 6c, where the *p*(*q*) of interfacial liquid water (blue curve) peaks at smaller *q*-values than that of the interfacial ice (violet and magenta), because on ice the tetrahedral ordering is stronger and, thus, *q*-values are closer to unity. Interestingly, consistent with the trends observed in the NEXAFS spectra, RES and ORC induce a local ordering of water in the interfacial region (red and green line, respectively), as shown by the shoulders of *p*(*q*) at values closer to *q*_t_, which is the threshold discriminating between liquid-like and ice-like water molecules. Moreover, interfacial water molecules are more ordered in the ORC solution than in the RES one, even at low concentration (0.2 M): this is ascribed to the fact that RES shows higher preference for the bulk and subsurface region than ORC (see Fig. 4), thus perturbing the ordering of water molecules at the interface. In addition, ORC tends to self-stack at the interface, likely forcing liquid water ordering by interaction among the ORC hydroxyl groups and water, as shown in Fig. 5.

The formation of a more ordered interfacial water layer at the RES and ORC aqueous solutions, somehow resemble those observed at the organic crystal/water interface,^9,107^ especially at phloroglucinol dihydrate crystals.^9^ At the water organic/crystal interface the 2D ordered distribution of hydrophilic (*i.e.*, –OH) and hydrophobic groups promotes water ordering.^9^ Here, due to the lower interfacial concentration of organics compared to that at crystal substrates, we do not observe a stable 2D pattern of adsorbates, but formation of islands with a self-assembled array of ORC (or RES) that dynamically form, disassemble and reform during the simulations. The hydrophobic (π–π) interaction among solutes orders the adsorbates at the interface, and this self-aggregation increases the local interfacial concentration, as shown in Fig. 5. This drives the interaction between the hydroxyl groups of the solutes and water to the formation of a more ordered interfacial water layer: this is evident in Fig. 6c where the tetrahedral order distribution is more shifted to higher *q*-values with RES and ORC in solution (green and red line), than on pure liquid water (blue line). Fig. S7,† which reports the distribution of the *q* order parameter as a function of the coordinate perpendicular to the interface, also supports the formation of a more ordered interfacial water layer in presence of ORC and RES. Our classical MD simulations report clear evidence of RES and ORC stacking at the interfaces, and similar features were also observed for other aromatic compounds in our previous works on the ice pre-melted liquid water surfaces.^97,98^ However, as we also remarked above, a more quantitative analysis of the adsorbate self-assembly, which drives the interfacial water ordering in ORC and RES aqueous solutions, calls for a better description of the π–π interaction, which is beyond the capability of the classical MD employed here and more suited for some *first-principle* MD approach.

### 3.3 Hydrogen bonding structure from valence spectra

**Fig. 7:**
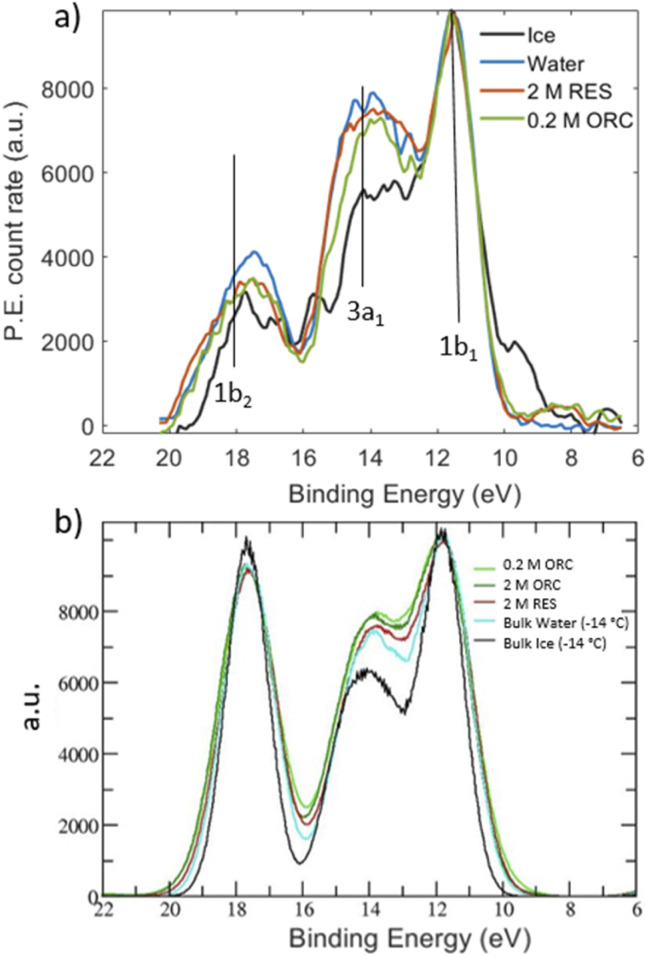
(a) Valence X-ray photoemission spectra for water (blue), ice (black), 2.0 M RES (red) and 0.2 M ORC, excited with a photon energy of 600 eV. The peaks labeled by 1b_1_, 3a_1_ and 1b_2_ correspond to the valence orbitals of the water molecules. All spectra are Shirley background removed and normalized with respect to their 1b_1_ peak height. The binding energy was fixed with respect to the 1b_1_ binding energy of liquid water. (b) DOS of liquid water molecules present in ORC, RES, bulk ice and bulk water. Data are aligned and normalized consistently with the experimental spectra. The DOS for the ORC and RES solutions show only the water contribution, *i.e.*, the solute DOSs were removed from the DOS of the entire system.

Inspired by the work of Winter *et al.*,^49^ we measured valence photoemission spectra of the solutions investigated in this work with the aim of finding a correlation between the occupied water molecular orbitals and the local hydrogen bond order. Fig. 7 shows valence level spectra in the region of the 1b_1_, 3a_1_ and 1b_2_ molecular orbitals of H_2_O for 2.0 M RES and 0.2 M ORC, together with those for liquid water (blue) and solid ice (black), normalized by the maximum intensity of the 1b_1_ peak. The gas phase contribution to the photoemission signal from the liquid–vapor interface has been subtracted from these spectra. Raw spectra are shown in the ESI (Section 5, Fig. S9†). Comparing the reference spectra of liquid water and ice, we observe that the 3a_1_ peak in ice (black) is lower than that of liquid water (blue), consistent with other literature reports.^55–57^ The 1b_2_ peak, which is also sensitive to the HB environment and orientation, also follows this trend.^50^ In presence of 2.0 M RES and 0.2 M ORC, both 3a_1_ and 1b_2_ tend to remain below those of liquid water, consistent with the presence of more ordered water, but still far away from that of ice. The noise level of the spectra does not allow making a statistically significant statement. While longer acquisition times would increase the signal-to-noise-ratio, spatial fluctuations of the liquid filament lead to increasing variability, also in terms of the fraction of gas phase contribution to the signal. The photoelectron kinetic energy range of the spectra in Fig. 7 corresponds to a mean escape depth of *ca.* 1.3 nm, or 4 monolayer-equivalents of water. From the depth profile characterization in Section 3.1, we note that organic molecules form a monolayer that accumulates at the interface of the RES and ORC solutions, with a thickness of about 2.2 Å (Fig. 3). Therefore, valence level spectra reported in Fig. 7a are contributed by both water molecules in close contact with organic molecules and water molecules further away in the bulk. The latter are less influenced by the presence of organic molecules, leading to a weakening of the signal, similar to the case of the NEXAFS spectra.

The theoretical DOS (Fig. 7b) reproduces the decrease of the 3a_1_ orbital in ice compared to that in liquid water, but basically no difference for the 1b_2_ peaks in comparison to liquid water. As benchmark test, Fig. S8† shows the DOS for a pure (*i.e.*, with no solutes) TIP4P/2005 bulk water and liquid water slab at 300 K, calculated by averaging the snapshots from the classical MD trajectories: the agreement with the bulk water DOS calculated over frames from a SCAN *first-principles* MD and reported in the original SCAN ref. 85 is quite remarkable, with the only slight overestimation of the 1b_2_ peak for the DOS from classical MD frames compared to that from the SCAN MD simulation. This further states the ability of TIP4P/2005 in describing the HB network in water and the possibility to have reliable configurations for DOS calculations at the convenient computational price of classical MD. We note that the calculated DOS in Fig. 7b displays the contributions from the water molecules only, after the removal of the RES or ORC DOS. Such a subtraction was not possible for the experimental spectra. Moreover, Fig. 7b shows a negligible impact of RES and ORC in the theoretical DOS, compared to the pure water case (cyan line). In view of the uncertainties in both experimental and theoretical spectra we refrain from making firmer conclusions about water ordering from the valence spectra. Further developments should include depth resolved measurements (with varying kinetic energy) and depth resolved calculations.

## 4. Conclusions

By using *in situ* XPS, we have experimentally investigated the surface excess and preferential orientation of RES and ORC molecules at the liquid–vapor interface. The surface excess was obtained by applying an attenuation model to kinetic energy dependent photoemission intensity measurements. The experimental results are in line with our classical MD simulations of RES and ORC at the aqueous interface, which further suggest self-aggregation *via* π-stacking. In addition, the O K-edge NEXAFS spectra of these organic aqueous solutions reveal a trend towards stronger ordering of water molecules at the liquid–vapor interface. This agrees with our calculation of the tetrahedral order parameter *q*, delivered by the MD simulations and suggests that the presence of resorcinol and orcinol molecules locally induce a more tetrahedral coordination among water molecules. Finally, measured valence level spectra also show a trend pointing towards more water ordering, though not at a statistically significant level. Similarly, the calculated DOS show only very small differences between liquid water and the solutions containing orcinol or resorcinol.

While our results do not directly provide parameters to be used in models treating ice nucleation in the atmosphere, our findings further our understanding of the basis of heterogeneous ice nucleation by organic compounds, specifically by aromatic compounds with OH substitution. Considering the ubiquitous presence of a broad range of OH substituted aromatic compounds in the atmosphere, their role in ice nucleation should be explored further in experiment and theory.

## Author contributions

H. Y., L. A. and M. A. designed the work and conceived the laboratory experiment; H. Y., A. B. and L. A. performed the XPS and NEXAFS spectroscopy measurements; H. Y. analysed and interpreted the XPS and NEXAFS data; I. G. contributed the computational part of the paper; H. Y., I. G., L. A. and M. A. worked on the manuscript together. All authors contributed to the discussion and approved the final version of the manuscript.

## Conflicts of interest

There are no conflicts to declare.

## Supplementary Material

EA-002-D2EA00015F-s001
