# Supplementary material for: Orcinol and resorcinol induce local ordering of water molecules near the liquid–vapor interface
Source: Environ Sci Atmos. 2022 Aug 23;2(6):1277–91. doi: 10.1039/d2ea00015f (PMC9648629; doi:10.1039/d2ea00015f)
Supplement: EA-002-D2EA00015F-s001 [file EA-002-D2EA00015F-s001.pdf]

Electronic Supplementary Material (ESI) for Environmental Science: Atmospheres.
This journal is © The Royal Society of Chemistry 2022

Orcinol and Resorcinol Induce Ordering of Water Molecules near the Liquid-Vapor Interface

Electronic Supporting Information

Huanyu Yang[a,b], Ivan Gladich[c], Anthony Boucly[a,d], Luca Artiglia[a,e], and Markus Ammann[a]*

a. Laboratory of Environmental Chemistry, Paul Scherrer Institut, 5232 Villigen, Switzerland.

b. Institute of Atmospheric and Climate Science, ETH Zürich, 8092 Zürich, Switzerland.

c. Qatar Environment & Energy Research Institute, Hamad Bin Khalifa University, P.O. Box 34110, Doha, Qatar.

d. Electrochemistry Laboratory, Paul Scherrer Institut, 5232 Villigen, Switzerland

e. Laboratory for Catalysis and Sustainable Chemistry, Paul Scherrer Institut, 5232 Villigen, Switzerland.

1. Schematic illustration of XPS configuration

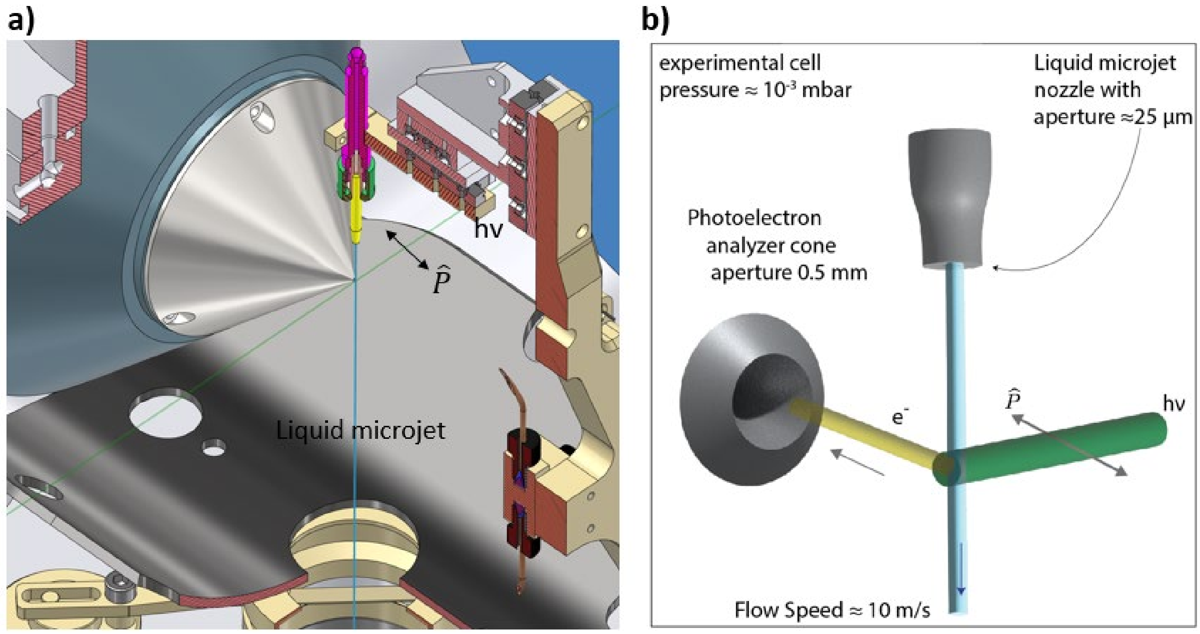

Figure S1: a) Sectional view of the liquid-gas analysis cell with the liquid microjet flowing downwards. b) Schematic illustration of the measurement configuration of the photoemission experiment. The excitation photon beam (green) is polarized such that its polarization vector ($\hat{P}$) is parallel to the photoelectron (yellow) detection direction. The downward travelling liquid filament (light blue), is orthogonal to the plane defined by the excitation photon beam and the photoelectron detection axis, which are also orthogonal to each other.

2. XPS of O 1s for RES and ORC at 155eV Kinetic Energy

In **Figure S2** we show an example of the O 1s photoemission signal, from 2.0 M RES and 0.2 M ORC solutions. The peak related to O 1s from the liquid phase is at binding energy 537.8 eV (green peak). A concomitant peak with a narrower FWHM, originating from the gas phase water appears near the liquid phase water O 1s (blue peak), shifted by 2.2 eV in binding energy. As discussed in the main text, 2.0 M RES and 0.2 M ORC, have similar surface excess. We do

not explicitly fit the small contribution of oxygens of the hydroxyl groups of RES and ORC, as H_2O largely dominates.

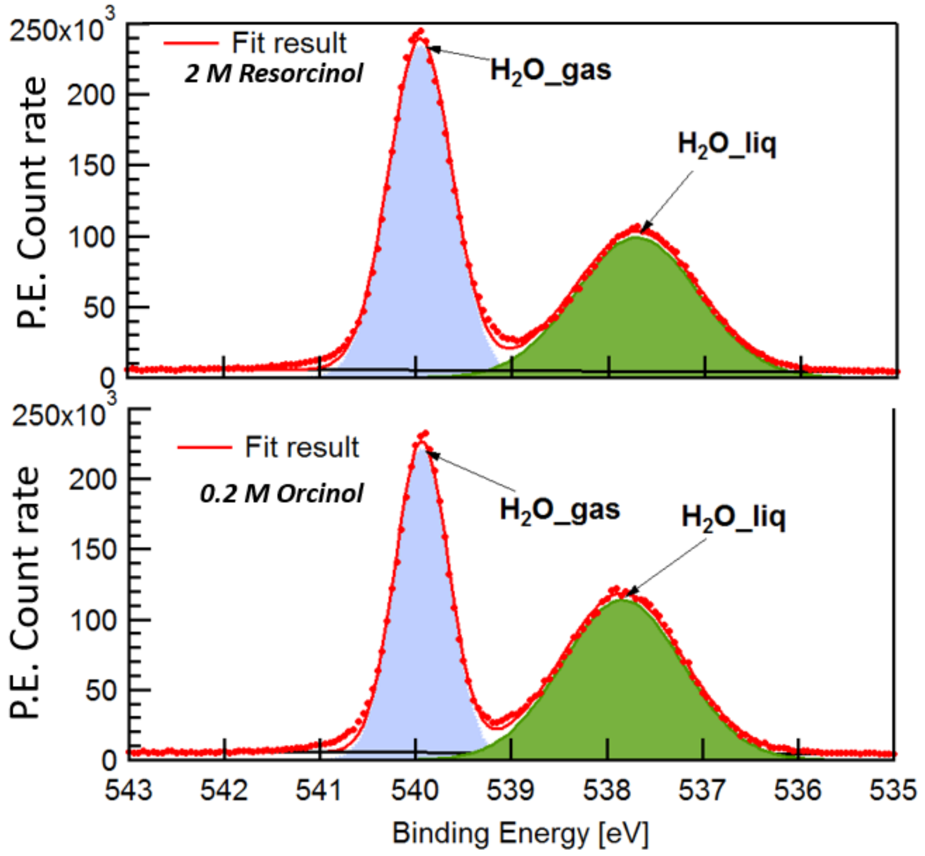

Figure S2: O 1s photoemission count rate of 2.0 M RES (top panel) and 0.2 M ORC (bottom panel) solutions. The excitation photon energy is 695 eV, so that the corresponding kinetic energy is ca. 155eV. The green peak represents the O 1s signal from water in the liquid phase, whereas the blue peak is assigned to gas phase water molecules with higher binding energy with respect to that in condensed phase. Both panels share the same vertical scale.

3. Attenuation model for the analysis of XPS signals

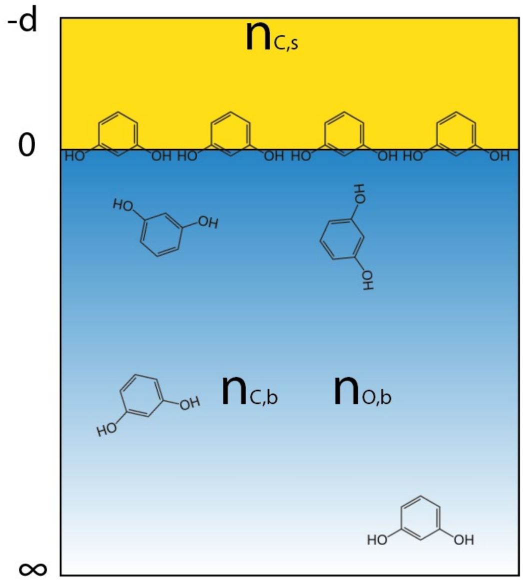

Figure S3: The attenuation model for determining the surface coverage for both resorcinol and orcinol solutions. We assume the organic monolayer establishes a homogeneous layer on the surface of the solution with thickness d (yellow shaded). Within this layer, the carbon atomic density is $n_{C,s}$, the atomic density of carbon and oxygen in the bulk (shaded in blue) are denoted as $n_{C,b}$ and $n_{O,b}$, respectively.

Figure S3 shows the conceptual model to assess attenuation of photoelectrons by adsorbed organic molecules. We assume the organic monolayer establishes a homogeneous layer on the surface of the solution with thickness d (shaded in yellow). Within this layer, the carbon atomic density is $n_{C,s}$ (in units of atoms cm^{-3}, numerical values are reported in **Table S1**). In the bulk solution phase below this layer (shaded in blue), the atomic density of carbon and oxygen are denoted as $n_{C,b}$ and $n_{O,b}$, respectively. Thus, for assessing the attenuation, we assume a homogeneous overlayer, for which we can estimate the inelastic mean free path, instead of using detailed atomic density profiles as returned from the MD simulation and electron scattering calculations on those. We assume the density of RES and ORC in this layer to be the same as that of their pure condensed phase (1.28 g/cm^3 and 1.29 g/cm^3 respectively[1, 2]), leading

to $n_{C,s}$ values of $4.20 \times 10^{22} cm^{-3}$ and $4.38 \times 10^{22} cm^{-3}$ for resorcinol and orcinol respectively. The C 1s photoemission intensity originating from molecules residing on the surface (and thus originating from carbon atoms contained in the yellow layer), $I_{C,s}$, is obtained by a simple attenuation law with mean escape depth $\frac{2}{\pi}\lambda_s$:

$$I_{C,s} = A \cdot \int_{-d}^{0} n_{C,s} \cdot e^{-\frac{z+d}{\frac{2}{\pi}\cdot\lambda_s}} dz = A \cdot n_{C,s} \cdot \frac{2}{\pi} \cdot \lambda_s \cdot \left(1 - e^{-\frac{d}{\frac{2}{\pi}\cdot\lambda_s}}\right)$$ Equation S1

The factor $A = \phi(hv, C1s) \cdot T \cdot \sigma_{T,C1s}(hv, \psi)$ contains the following quantities: $\phi(hv, C1s)$ is the excitation photon flux (number of photons per second) with photon energy hv used for the measurement of the C 1s spectrum for a given kinetic energy. $T = \Omega_0 \cdot A_0 \cdot D_0$ is a factor combines electron detection efficiency (D_0), the acceptance angle (Ω_0) and the effectively analyzed area (A_0), where the latter two are depending on the geometry of our experimental setup, while the first is depending on the kinetic energy. T is independent of the sample or photon energy selected, but depending on the kinetic energy. When calculating the C/O photoemission signal intensity ratios at the same KEs (see below), this factor cancels.

$\sigma_{T,C1s}(hv)$ is the total differential photoionization cross section (Mbarn) for the C 1s core level, expressed as:

$$\sigma_{T,C1s}(hv, \psi) = \sigma_{C1s}(hv) \cdot \frac{1 + \beta(hv) \cdot P_2(\cos(\psi))}{4\pi}$$ Equation S2

In Equation S2, $\beta(hv)$ is defined as the asymmetry parameter. P_2 is the second order Legendre Polynomial, as in our experiment setup $\psi = 0$, thus $P_2(\cos(\psi)) = 1$. $\sigma_{C1s}(hv)$ is the excitation energy dependent photoionization cross section of the C 1s core-electron. In the attenuation model described in Equation S1, we consider only the inelastic scattering of photoelectrons. The elastic scattering effect is very weak and can be neglected in our system.

In the bulk, the O 1s photoemission signal is predominantly originating from oxygen of bulk water molecules. The surface excess of the 0.2 M orcinol and the 2.0 M resorcinol solutions are about the same (see below). Both molecules contain one oxygen atom in each hydroxyl group. The fraction of H_2O-oxygen within the topmost water bilayer is three and one, respectively, orders of magnitude higher than the fraction of organic hydroxyl group oxygen. So, we neglect the atomic density of organic OH-group oxygen at the surface, considering only that of the water in the bulk, as $n_{O,b}$ (constant value of $3.45 \cdot 10^{22}\ cm^{-3}$). The photoemission intensity of oxygen and carbon from the bulk (denoted as $I_{C,b}$ and $I_{O,b}$, with atomic density $n_{C,b}$ and $n_{O,b}$, respectively), is obtained by integrating z from 0 to ∞, with the attenuation factor $e^{-\frac{d}{\frac{2}{\pi}\cdot\lambda_s}}$ due to organic overlayer:

$$I_{C,b} = A \cdot \int_0^{\infty} n_{C,b} \cdot e^{-\frac{z}{\frac{2}{\pi}\cdot\lambda_b}} dz \cdot e^{-\frac{d}{\frac{2}{\pi}\cdot\lambda_s}} = A \cdot n_{C,b} \cdot \frac{2}{\pi} \cdot \lambda_b \cdot e^{-\frac{d}{\frac{2}{\pi}\cdot\lambda_s}}$$ Equation S3

And similarly for $I_{O,b}$:

$$I_{O,b} = B \cdot n_{O,b} \cdot \frac{2}{\pi} \cdot \lambda_b \cdot e^{-\frac{d}{\frac{2}{\pi}\cdot\lambda_s}}$$ Equation S4

where B is the factor with same structure as A used in Equation S1, but parameterized exclusively for the detection of O 1s: $B = \phi(hv, O1s) \cdot T \cdot \sigma_{T,O1s}(hv, \psi)$. The atomic density of carbon in the bulk, $n_{C,b}$, depends on the solution concentration, listed in **Table S1**. The experimentally determined overall total C 1s photoemission intensity $I_{C,T}$ can be compared to the calculated sum of $I_{C,s}$ and $I_{C,b}$. The total condensed phase O 1s photoemission intensity $I_{O,T}$ is equal to $I_{O,b}$.

As discussed in the previous section, because resorcinol and orcinol molecules are surface active, we expect an enrichment of these organic molecules on the solution surface, and lower

concentration in the bulk. This is apparent from the $I_{C,T}/I_{O,T}$ photoemission signal intensity ratio as a function of photoelectron kinetic energy, referred to as normalized C/O depth profile. When computing the normalized C/O depth profile, by combining Equations S1-S3, we can write:

$$\frac{I_{C,T}}{I_{O,T}} \cdot \frac{B}{A} = \frac{I_{C,T}}{I_{O,T}} \cdot \frac{\phi(hv, O1s) \cdot \sigma_{T,O1s}(hv, \psi)}{\phi(hv, C1s) \cdot \sigma_{T,C1s}(hv, \psi)}$$

Equation S5

$$= \frac{n_{cb}}{n_{ob}} + \frac{n_{cs}}{n_{ob}} \cdot \frac{\lambda_s}{\lambda_b} \cdot \left(e^{+\frac{d}{\frac{2}{\pi}\lambda_s}} - 1 \right)$$

where λ_s and λ_b were estimated by the SESSA software and its corresponding database.[3] As noted above, the not well known transmission function, *T*, contained in both *A* and *B*, cancels. And thus, all parameters in equation S5 are known except the layer thickness, *d*.

The surface propensity of solutes at the liquid-vapor interface is described by the surface excess, Γ, which denotes the deviation of the solute density in the interfacial region compared that in the bulk. In our case, Γ is determined by the product of d and n_s, where n_s is the molecule number density within this layer on the surface, as introduced in the main text. The numerical value of n_s is derived from $n_{C,s}$, by the factor equivalent to the number of carbon atoms within the molecule. In our case, n_s is equal to $n_{C,s}/6$ and $n_{C,s}/7$ for resorcinol and orcinol, respectively. Numerical values are reported in **Table S1**.

Table S1: Quantities used in the attenuation model and the fitting result in terms of thickness d. The surface excess Γ is obtained by the product of n_s and d.

Sample	$n_{C,b}$ (atoms/cm^3)	$n_{C,s}$ (atoms/cm^3)	n_s (molecules/cm^3)	d (Å)	Γ (molecules/cm^2)
Resorcinol 0.01M	$3.61 \cdot 10^{19}$	4.20×10^{22}	6.84×10^{21}	0.085	5.8×10^{12}
Resorcinol 2M	$7.2 \cdot 10^{21}$	4.20×10^{22}	6.84×10^{21}	2.2	1.5×10^{14}
Orcinol 0.01M	$4.21 \cdot 10^{19}$	4.38×10^{22}	6.26×10^{21}	0.26	1.6×10^{13}
Orcinol 0.2M	$8.4 \cdot 10^{20}$	4.38×10^{22}	6.26×10^{21}	2.2	1.4×10^{14}

4. Molecular dynamics and Density of States Computational Details

4.1 Simulation Box preparation

A liquid water box of ~15 × 15 × 32 Å^3 consisting of 216 water molecules was equilibrated at 1 bar pressure and 300 K by running 1 ns classical MD using TIP3P [4] or TIP4P/2005[5] water model. Afterwards, the Z-dimension of the simulation box was enlarged to 72 Å and the system was further equilibrated during additional 1 ns at constant volume and temperature (i.e., the so called NVT ensemble) MD, finally resulting in a water slab system with two equilibrated vapor/liquid water interfaces. The simulation box size adopted here has been recognized to be sufficient in describing a liquid water slab with both well-defined interfacial and bulk solvation environments [6]. Very similar equilibration protocols at classical MD have been reported in the literature.[7, 8]

Starting from the equilibrated liquid water slab, three different initial solute concentrations were created. In the first two, 4 orcinol (or 4 resorcinol) molecules were placed on each of the two liquid water interfaces, resulting in a total of 8 orcinol (or 8 resorcinol) and an aqueous solution concentration of 2 M. The third configuration of 0.2 M orcinol was prepared by placing 1 orcinol on the water slab. These three systems were selected to model the experimental concentrations (i.e., 2 M resorcinol and 0.2 M orcinol) and to offer a molecular picture for the dynamics of the two compounds at the same (2 M) concentration. For each concentration, we performed two separate 400 ns NVT MD runs using the TIP3P or the TIP4P/2005 water model, for a total of six NVT simulations at 300 K. Figure S4 shows a snapshot from the MD trajectory of 2 M ORC at 300 K.

An additional ice slab of 320 TIP4P/2005 water molecules with two vapor/ice interfaces was prepared from an initial proton disordered crystal of hexagonal (I_h) ice of dimension ~ 1.8 nm x 16 nm x 3.7 using the Buch algorithm.[9] A 1.5 ns constant pressure simulation (i.e., NpT)

MD at 0 bar with a time step of 0.1 fs, followed by another 0.5 ns at the target temperature, was performed to anneal this ice crystal from 0 K to 237 K, which is 14 K below the melting temperature (Tm=251K) of TIP4P/2005.[10] Afterward, the Z-dimension of the simulation box was extended to 7.2 nm, resulting in an ice slab with two vapor-exposed basal ice facets. Starting from this equilibrated ice slab system, 400 ns NVT production runs were performed. Similar protocols for the preparation of ice slab simulations have been exploited successfully in the literature.[11-14] [15] An ice slab configuration taken from the MD trajectory at 237K is shown in Figure S6a.

4.2 Gibbs Dividing Surface and Solute Surface Excess

The Gibbs Dividing Surface (GDS) and the solute surface excess, Γ, have been determined following Ref. [16, 17] The excess number of a generic species at $z = 1$ is

$$N^{ex}(\mathrm{l}) = 2\mathrm{A}\Gamma(\mathrm{l}) = A\left(\int_{-l}^{l}(\rho_N - \rho_N^{bulk})dz + \int_{l}^{+\infty}(\rho_N - \rho_N^{gas})dz + \int_{-\infty}^{-l}(\rho_N - \rho_N^{gas})dz\right) \qquad \text{Equation S6}$$

Where z is the coordinate perpendicular to the interface. The factor 2 on the right-hand side of equation S6 accounts for the presence of two vapor/liquid water interfaces (Figures S4-S5). Γ is the surface excess in molecules/nm^2. ρ_N (in molecules/nm^3) is the number density and its integration over the whole simulation box (considering that our slab system is homogeneous in x and y) yields

$$A \cdot \int_{-\infty}^{+\infty} \rho_N dz = \mathrm{N} \qquad \text{Equation S7}$$

the number, N, of solute (or solvent). ρ_N^{Bulk} and ρ_N^{gas} are the number density in the solution bulk and in the gas phase, respectively. A is the area of the interface (= 1.48×1.48 nm^2 for our water slab system) perpendicular to the z-direction.

Equation S7 can be simplified considering that, in our case, ρ_N^{gas}=0 and ρ_N^{Bulk} is constant

$$\Gamma(l) = \frac{1}{2}\left(\int_{-\infty}^{+\infty} \rho_N dz - 2l\rho_N^{bulk}\right)$$ Equation S8

The density profiles in Figure S5 are expressed as $\rho = n_i/n_{TOT}$, which are normalized to the unity, i.e.,

$$\rho_N = \frac{N}{A}\rho = \frac{N}{A}(n_i/n_{TOT})$$ Equation S9

In terms of ρ, Equation S9 becomes

$$\Gamma(l) = \frac{N}{2A}(1 - 2l\rho^{bulk})$$ Equation S10

The Gibbs dividing surface (GDS) is defined as the surface of zero-solvent excess, i.e.,

$$Z_{GDS} = l = \frac{1}{2\rho^{wat,bulk}}$$ Equation S11

where $\rho^{wat,bulk}$ is the values of the water density profile in Figure S5 in the bulk (i.e., $z = 0$). The solute surface excess at the GDS is

$$\Gamma = \Gamma_{solute}(Z_{GDS}) = \frac{N}{2A}(1 - \frac{\rho^{solute,bulk}}{\rho^{wat,bulk}})$$ Equation S12

where $\rho^{sol,bulk}$ is the value of the solute density profile in Figure S5 in the bulk (i.e., $z = 0$).

The bulk concentration is defined as number of molecules / nm^3 in the solution bulk within the two GDS, i.e.,

$$n_b = \frac{1}{Volume}\int_{-ZGDS}^{ZGDS} \rho_N^{bulk}\, dz = \frac{\rho_N^{sol,bulk}}{A} = \frac{N\rho^{sol,bulk}}{A^2}$$ Equation S13

4.3 Molecular Dynamics Force Field and Simulations details

The generalized AMBER force field, GAFF2, practice[18] was adopted to create the force field parameters for orcinol and resorcinol. Molecular structures were optimized at the MP2/6-31G* level of theory and atomic partial charges were determined by the Restrained Electrostatic Potential (RESP) method with a Merz-Singh-Kollman scheme,[19] fitting the electrostatic potential obtained from single point energy calculations at the HF/6-31g* level on the MP2-optimized structures. Gaussian09[20] and Antechamber[21] were employed to perform the optimization and the charge fitting procedure, respectively. Torsion and non-bonded Lennard-Jones parameters were taken from GAFF2. This, or a very similar, force field strategy has been successfully used in the description of different aqueous systems, even in the presence of heterogeneous environments, such as vapor/liquid water interfaces.[7, 22]

The GROMACS 2018.6[23] molecular dynamics package was employed to run all the classical MD simulations, employing the leap-frog integration algorithm[24] and a time step of 2 fs. The Lennard-Jones potential and the real part of the Coulomb interactions were truncated at 0.7 nm. The long-range part of the electrostatic and Lennard-Jones interactions were treated by the particle mesh Ewald method,[25, 26] using a relative tolerance of 10^{-5}, fourth-order cubic interpolation, and a Fourier spacing parameter of 0.12. A stochastic velocity rescaling thermostat[27], with a time constant of 0.1 ps was used to control the temperature. The Berendsen barostat[28] with a time constant of 2ps was employed to control the pressure during the NpT equilibration runs of the liquid water slab. The SETTLE algorithm[29] was used to constrain the TIP4P/2005 water geometry, and the LINCS algorithm[30] was exploited to constrain covalent bonds involving hydrogen atoms in orcinol and resorcinol molecules. PLUMED 2.5 [31] was also used for the post-processing analysis.

Table S2: The solute surface excess at the GDS (*Γ*) and the bulk concentrations (n_b) obtained from the density profiles reported in Figure S5 for the different water models.

	Γ<br>(10^{14} molecules/cm^2)	n_b<br>(10^{21} molecules/cm^3)
0.2 M ORC, TIP3P	1.2	0.18
0.2 M ORC, TIP4P/2005	1.8	0.00
2 M ORC, TIP3P/2005	1.4	0.12
2 M ORC, TIP4P/2005	1.8	0.01
2M RES, TIP3P	0.8	0.28
2M RES, TIP4P/2005	1.6	0.06

Table S3: Comparison between experimental and computational solute surface excess (*Γ*) and bulk solute concentration, n_b. Computational values refer to those in TIP3P water. Experimental values are taken from Table S1

	Experimental		Computational	
Sample	$\Gamma(cm^{-2})$	$n_b\ (cm^{-3})$	$\Gamma(cm^{-2})$	$n_b\ (cm^{-3})$
0.01 M RES	$5.8 \cdot 10^{12}$	$0.60 \cdot 10^{19}$	/	/
2 M M RES	$1.5 \cdot 10^{14}$	$1.2 \cdot 10^{21}$	$0.8 \cdot 10^{14}$	$0.28 \cdot 10^{21}$
0.01 M ORC	$1.6 \cdot 10^{13}$	$0.60 \cdot 10^{19}$	/	/
0.2 M ORC	$1.4 \cdot 10^{14}$	$1.2 \cdot 10^{20}$	$1.2 \cdot 10^{14}$	$1.8 \cdot 10^{20}$
2 M ORC	/	/	$1.4 \cdot 10^{14}$	$1.2 \cdot 10^{20}$

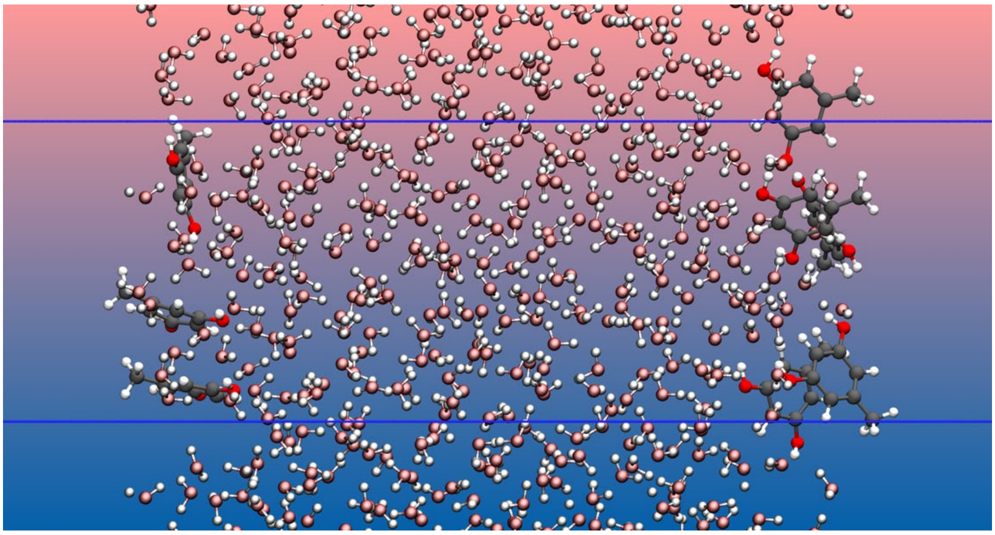

Figure S4: A snapshot from the 2M ORC MD simulation in TIP3P water at 300 K. In metal pastel the water molecules. Color code for the organic: H (white), O (red), C (grey).

	TIP3P	TIP4P/2005
0.2M ORC	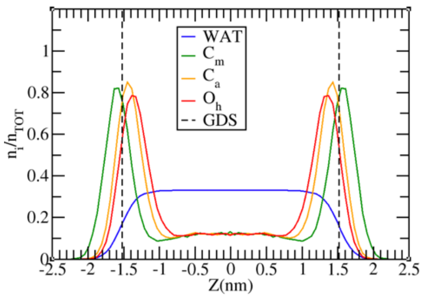	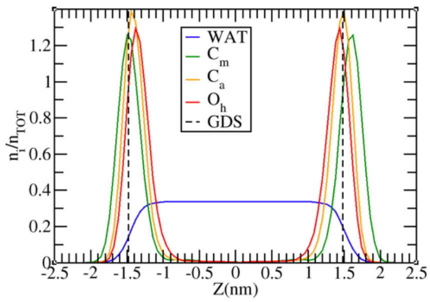
2M ORC	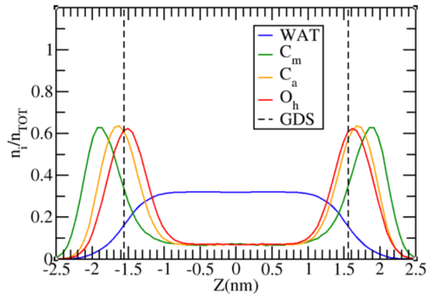	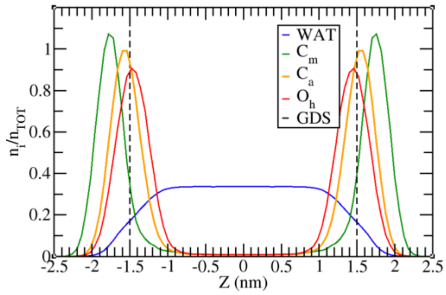
2M ORC Larger system (24 ORC In 5400 WATER)		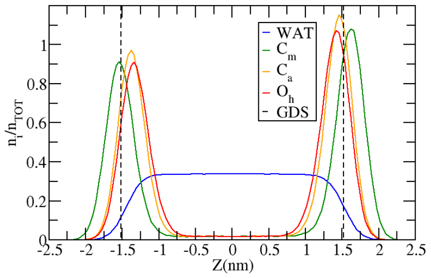
2M RES	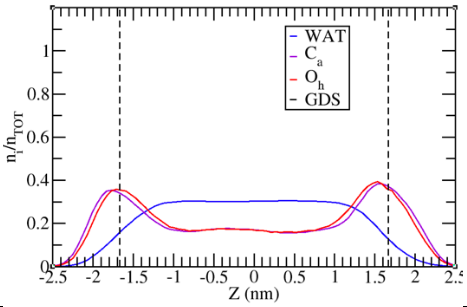	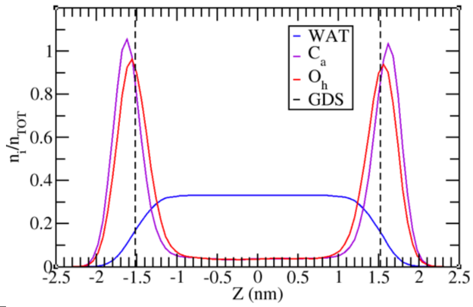

Figure S5: The probability distribution profile, n_i/n_{TOT}, normalized to unity as a function of the z-coordinate perpendicular to the vapor/liquid water interface, obtained from collecting the z-position of each species in the different aqueous solutions (first column) using a TIP3P water (second column) and a TIP4P/2005 water slab (third column). Top panels report the water model used for the MD simulation.

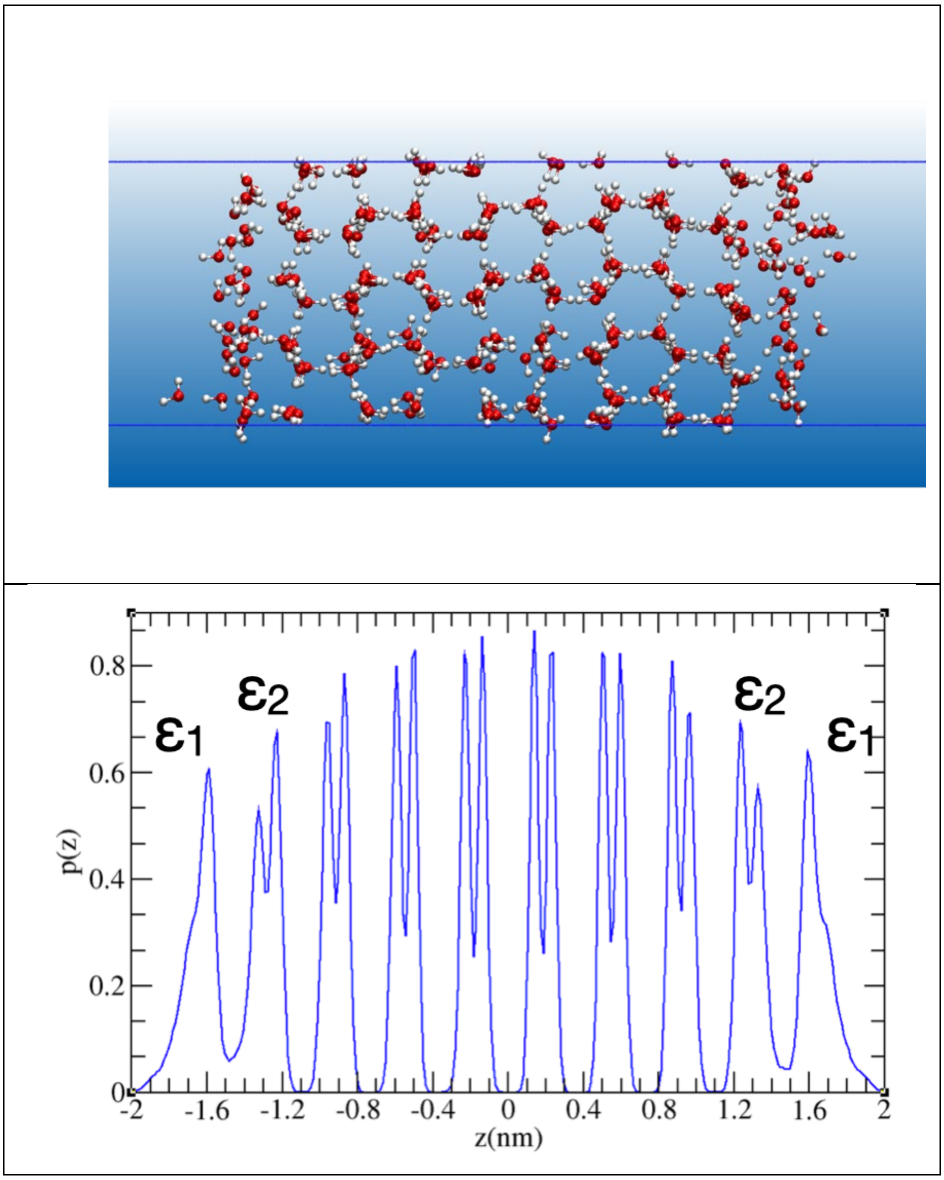

Figure S6: Top panel: A snapshot taken from the MD trajectory of the ice slab at 237 K, showing the two vapor/ice interfaces and the simulation box (in blue). The snapshot shows a view of the secondary prismatic facet, while the interfaces exposed to the vapor phase are the basal ones. Lower panel: the probability distribution profile, p(z), as a function of the z-coordinate perpendicular to the vapor/ice interface. p(z) has not been normalized to units for visual purpouses. ε_1 and ε_2 denote the interfacial bi-layers closer to vapor interface. [32]

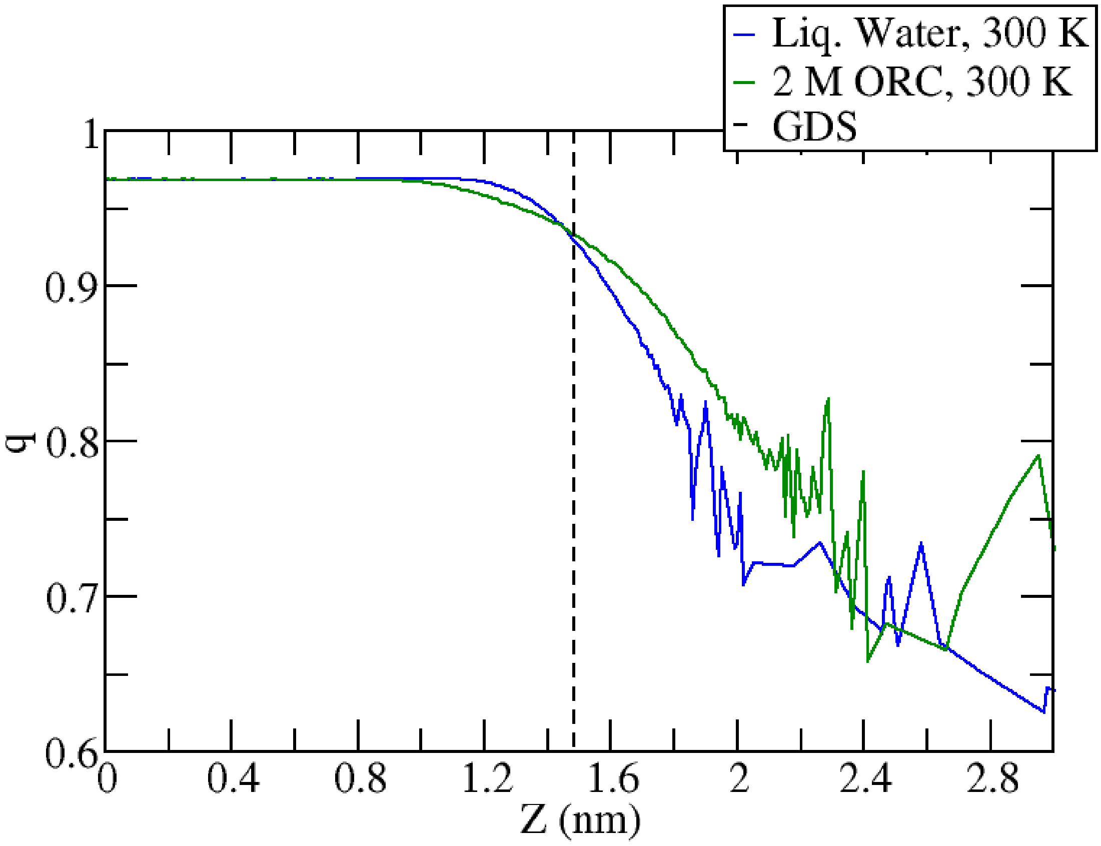

Figure S7: The distribution of the order parameter, q, as a function of the coordinate perpendicular to the interface, Z. $Z = 0$ corresponds to the bulk region. At the interface, defined as Z larger than the Gibbs Dividing Surface, the order distribution of liquid water is higher for the 2 M ORC solution (green line) than for the pure liquid water one (blue line), in agreement with the conclusions from Figure 6c of the main text.

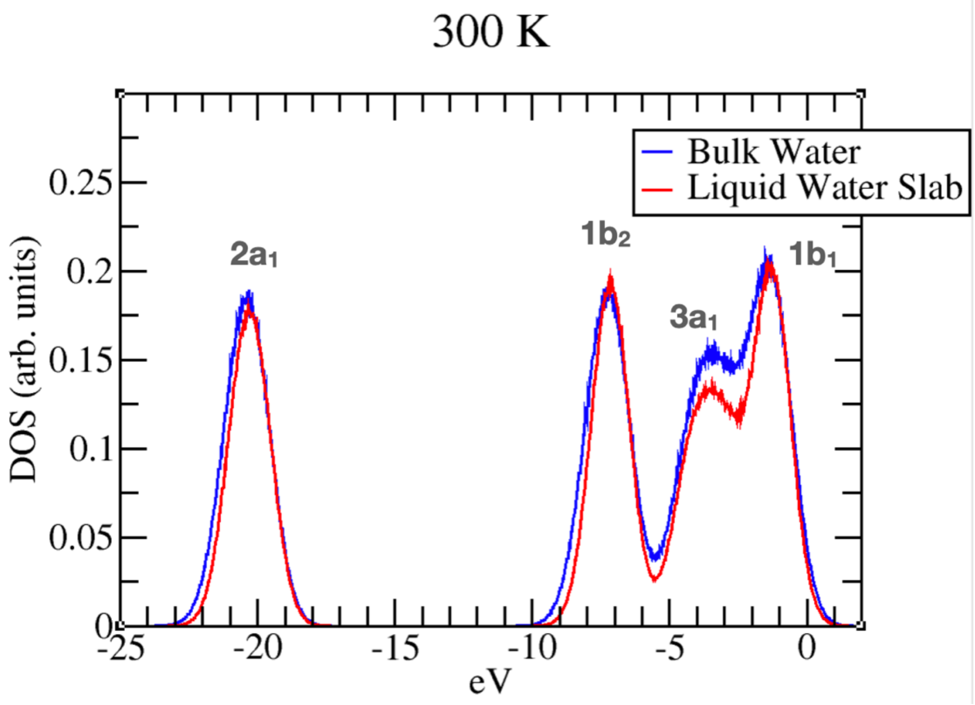

Figure S8: DOS at 300 K of bulk liquid water (blue) and liquid water slab (red) calculated at SCAN DFT level over snapshots taken from the classical MD trajectories using TIP4P/2005. Data are aligned and normalized to the O $1b_1$ peak of bulk water. A Gaussian smearing of 0.5 eV was used to smooth the DOS.

5. Raw valence level spectra with gas phase water molecules contribution

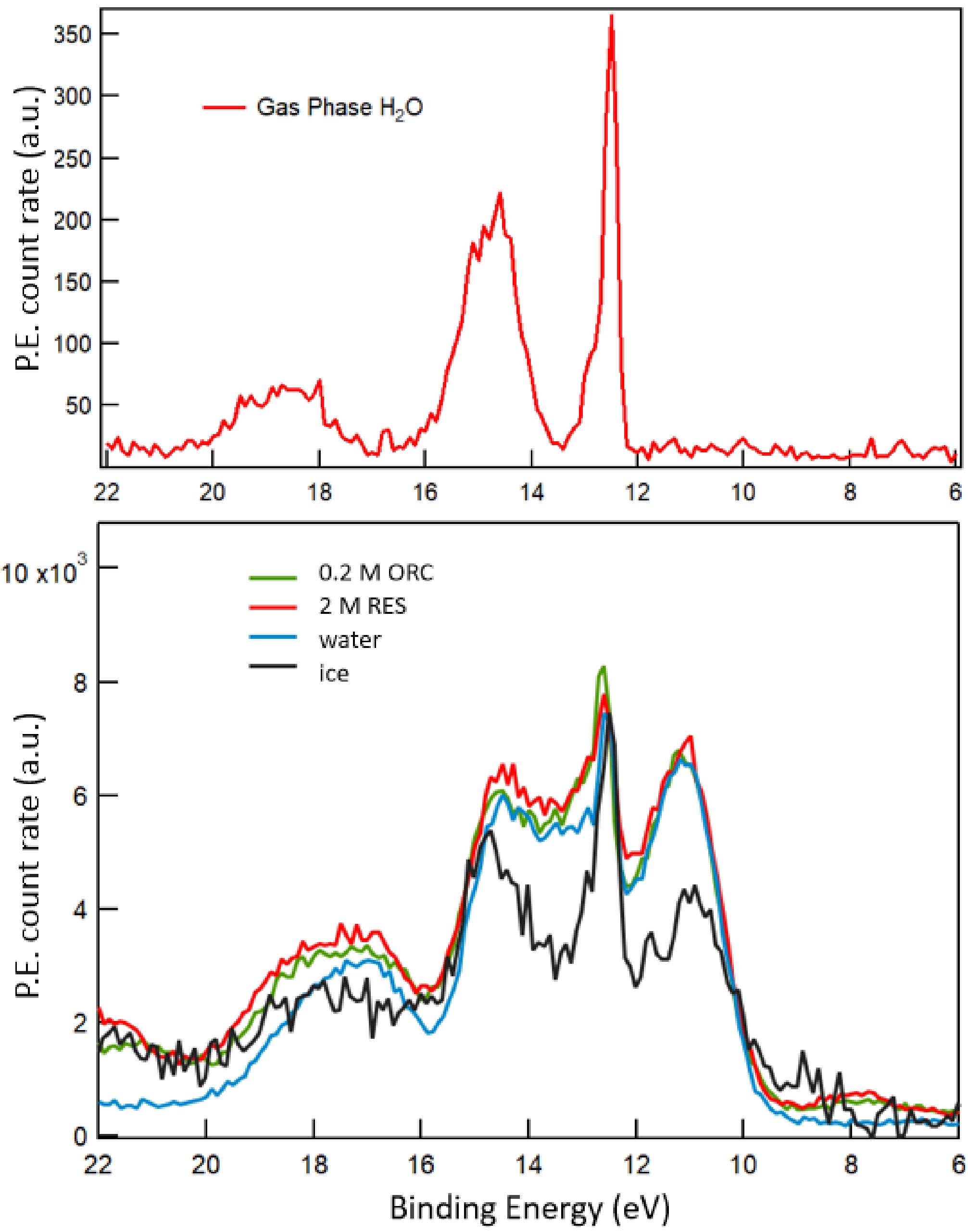

Figure S9: Upper panel: Valence level spectra of gas phase water molecules. Bottom panel: valence level without removing the gas phase contribution for water (blue), ice (black, -15 °C), 2M RES (red) and 0.2 M ORC (green), with excitation photon energy 600eV.

REFERENCES

1. CSID:4878, http://www.chemspider.com/Chemical-Structure.4878.html, (accessed Aug 11, 2022, 2022).
2. CSID:13839080, http://www.chemspider.com/Chemical-Structure.13839080.html, (accessed Aug 11, 2022, 2022).
3. C. J. Powell, A. Jablonski and F. Salvat,NIST databases with electron elastic-scattering cross sections, inelastic mean free paths, and effective attenuation lengths *Surface and Interface Analysis: An International Journal devoted to the development and application of techniques for the analysis of surfaces, interfaces and thin films*, 2005, **37**, 1068-1071.
4. W. L. Jorgensen, J. Chandrasekhar, J. D. Madura, R. W. Impey and M. L. Klein,Comparison of simple potential functions for simulating liquid water *J. Chem. Phys*, 1983, **79**, 926-935.
5. J. L. Abascal and C. Vega,A general purpose model for the condensed phases of water: TIP4P/2005 *J. Chem. Phys*, 2005, **123**, 234505.
6. I.-F. W. Kuo and C. J. Mundy,An ab Initio Molecular Dynamics Study of the Aqueous Liquid-Vapor Interface *Science*, 2004, **303**, 658-660.
7. I. Gladich, A. Habartová and M. Roeselová,Adsorption, mobility, and self-association of naphthalene and 1-methylnaphthalene at the water–vapor interface *J. Phys. Chem. A*, 2014, **118**, 1052-1066.
8. I. Gladich, A. Abotaleb and A. Sinopoli,Tuning CO2 Capture at the Gas/Amine Solution Interface by Changing the Solvent Polarity *J. Phys. Chem. B*, 2020, **124**, 10245-10256.
9. V. Buch, P. Sandler and J. Sadlej, *Journal*, 1998, **102**, 8641-8653.
10. R. García Fernández, J. L. Abascal and C. Vega,The melting point of ice I h for common water models calculated from direct coexistence of the solid-liquid interface *J. Chem. Phys*, 2006, **124**, 144506.
11. E. Muchova, I. Gladich, S. Picaud, P. N. Hoang and M. Roeselova,The Ice− Vapor Interface and the Melting Point of Ice I h for the Polarizable POL3 Water Model *J. Phys. Chem. A*, 2011, **115**, 5973-5982.
12. M. Conde, C. Vega and A. Patrykiejew,The thickness of a liquid layer on the free surface of ice as obtained from computer simulation *J. Chem. Phys*, 2008, **129**, 014702.
13. I. Gladich, W. Pfalzgraff, O. Maršálek, P. Jungwirth, M. Roeselová and S. Neshyba,Arrhenius analysis of anisotropic surface self-diffusion on the prismatic facet of ice *Phys. Chem. Chem. Phys.*, 2011, **13**, 19960-19969.
14. I. Gladich and M. Roeselová,Comparison of selected polarizable and nonpolarizable water models in molecular dynamics simulations of ice I h *Phys. Chem. Chem. Phys.*, 2012, **14**, 11371-11385.
15. I. Gladich, M. L. Berrens, P. M. Rowe, R. G. Pereyra and S. Neshyba,Solvation and stabilization of single-strand RNA at the air/ice interface support a primordial RNA world on ice *J. Phys. Chem. C* . 2020, **124**, 18587-18594.
16. L. Vrbka, M. Mucha, B. Minofar, P. Jungwirth, E. C. Brown and D. J. Tobias,Propensity of soft ions for the air/water interface *Curr Opin Colloid Interface Sci*, 2004, **9**, 67-73.

17. L. Herrera, C. Fan, D. D. Do and D. Nicholson,A revisit to the Gibbs dividing surfaces and helium adsorption *Adsorption*, 2011, **17**, 955-965.
18. J. P. M. Jämbeck and A. P. Lyubartsev,Update to the General Amber Force Field for Small Solutes with an Emphasis on Free Energies of Hydration *The Journal of Physical Chemistry B*, 2014, **118**, 3793-3804.
19. C. I. Bayly, P. Cieplak, W. Cornell and P. A. Kollman,A well-behaved electrostatic potential based method using charge restraints for deriving atomic charges: the RESP model *J. Phys. Chem.*, 1993, **97**, 10269-10280.
20. G. W. T. M. J. Frisch, H. B. Schlegel, G. E. Scuseria, M. A. Robb, J. R. Cheeseman, G. Scalmani, V. Barone, G. A. Petersson, H. Nakatsuji, X. Li, M. Caricato, A. Marenich, J. Bloino, B. G. Janesko, R. Gomperts, B. Mennucci, H. P. Hratchian, J. V. Ortiz, A. F. Izmaylov, J. L. Sonnenberg, D. Williams-Young, F. Ding, F. Lipparini, F. Egidi, J. Goings, B. Peng, A. Petrone, T. Henderson, D. Ranasinghe, V. G. Zakrzewski, J. Gao, N. Rega, G. Zheng, W. Liang, M. Hada, M. Ehara, K. Toyota, R. Fukuda, J. Hasegawa, M. Ishida, T. Nakajima, Y. Honda, O. Kitao, H. Nakai, T. Vreven, K. Throssell, J. A. Montgomery, Jr., J. E. Peralta, F. Ogliaro, M. Bearpark, J. J. Heyd, E. Brothers, K. N. Kudin, V. N. Staroverov, T. Keith, R. Kobayashi, J. Normand, K. Raghavachari, A. Rendell, J. C. Burant, S. S. Iyengar, J. Tomasi, M. Cossi, J. M. Millam, M. Klene, C. Adamo, R. Cammi, J. W. Ochterski, R. L. Martin, K. Morokuma, O. Farkas, J. B. Foresman, and D. J. Fox, *Gaussian 09, Revision A.02*, Gaussian, Inc., Wallingford CT, 2016.
21. J. M. Wang, W. Wang and P. A. Kollman,Antechamber: An accessory software package for molecular mechanical calculations *Abstracts of Papers of the American Chemical Society*, 2001, **222**, U403.
22. A. Habartová, K. T. Valsaraj and M. Roeselová,Molecular Dynamics Simulations of Small Halogenated Organics at the Air–Water Interface: Implications in Water Treatment and Atmospheric Chemistry *J. Phys. Chem. A*, 2013, **117**, 9205-9215.
23. M. J. Abraham, T. Murtola, R. Schulz, S. Páll, J. C. Smith, B. Hess and E. Lindahl,GROMACS: High performance molecular simulations through multi-level parallelism from laptops to supercomputers *SoftwareX*, 2015, **1-2**, 19-25.
24. R. W. Hockney, S. Goel and J. Eastwood,Quiet high-resolution computer models of a plasma *J. Comput. Phys.*, 1974, **14**, 148-158.
25. T. Darden, D. York and L. Pedersen,Particle mesh Ewald: An N· log (N) method for Ewald sums in large systems *J. Chem. Phys*, 1993, **98**, 10089-10092.
26. U. Essmann, L. Perera, M. L. Berkowitz, T. Darden, H. Lee and L. G. Pedersen,A smooth particle mesh Ewald method *J. Chem. Phys*, 1995, **103**, 8577-8593.
27. G. Bussi, D. Donadio and M. Parrinello,Canonical sampling through velocity rescaling *J. Chem. Phys*, 2007, **126**, 014101.
28. H. J. C. Berendsen, J. P. M. Postma, W. F. v. Gunsteren, A. DiNola and J. R. Haak,Molecular dynamics with coupling to an external bath *J. Chem. Phys*, 1984, **81**, 3684-3690.
29. S. Miyamoto and P. A. Kollman,Settle: An analytical version of the SHAKE and RATTLE algorithm for rigid water models *J. Comput. Chem.*, 1992, **13**, 952-962.
30. B. Hess, H. Bekker, H. J. Berendsen and J. G. Fraaije,LINCS: a linear constraint solver for molecular simulations *J. Comput. Chem.*, 1997, **18**, 1463-1472.
31. G. A. Tribello, M. Bonomi, D. Branduardi, C. Camilloni and G. Bussi,PLUMED 2: New feathers for an old bird *Comput. Phys. Commun.*, 2014, **185**, 604-613.

32. I. Gladich, A. Oswald, N. Bowens, S. Naatz, P. Rowe, M. Roeselova and S. Neshyba,Mechanism of anisotropic surface self-diffusivity at the prismatic ice–vapor interface *Phys. Chem. Chem. Phys.*, 2015, **17**, 22947-22958.
